# Purchaser, firearm, and retailer characteristics associated with crime gun recovery: a longitudinal analysis of firearms sold in California from 1996 to 2021

**DOI:** 10.1186/s40621-024-00491-8

**Published:** 2024-02-26

**Authors:** Sonia L. Robinson, Christopher D. McCort, Colette Smirniotis, Garen J. Wintemute, Hannah S. Laqueur

**Affiliations:** Violence Prevention Research Program, Department of Emergency Medicine, University of California, Davis, Sacramento, CA USA

**Keywords:** Firearm recovery, Weapons crime, Violent crime, Firearm dealerships, Firearm purchasers, Firearm transactions, Crime guns, Gun theft, Criminal gun markets

## Abstract

**Background:**

Firearm violence is a major cause of death and injury in the United States. Tracking the movement of firearms from legal purchase to use in crimes can help inform prevention of firearm injuries and deaths. The last state-wide studies analyzing crime gun recoveries used data from over 20 years ago; thus, an update is needed.

**Methods:**

We used data for 5,247,348 handgun and 2,868,713 long gun transactions and law enforcement recoveries from California crime gun recovery (2010–2021) and California’s Dealer Records of Sales records. Covariates included characteristics of dealership sales, firearms and their transactions, and purchaser’s demographic characteristics, purchasing history, criminal history (from firearm purchaser criminal history records), and neighborhood socioeconomic status. Analyses for handguns and long guns was conducted separately. In multivariable analysis, we included correlates into a Cox proportional hazard model accounting for left truncation and clustering between the same firearm, purchaser, dealerships, and geographic location. Covariates that remained significant (*P* < 0.05) were retained. For handguns, we evaluated associations of violent and weapons crimes separately. In supplementary analyses, we examined interactions by purchasers’ race and ethnicity.

**Results:**

In total, 38,441 handguns (0.80%) and 6,806 long guns (0.24%) were recovered in crimes. A firearm dealer’s sales volume, percent of transactions that were denials, pawns, pawn redemptions, and firearms that became crime guns were each positively associated with firearm recovery in crime. Handguns that were inexpensive, larger caliber, and that had been reported lost or stolen were positively associated with recovery in crimes. Purchaser characteristics associated with crime gun recovery included: being younger, female, Black, Hispanic, Native American or Pacific Islander, or other race/ethnicity (vs white), having previous arrests, living in close proximity to the firearm dealership, and living in a more socially vulnerable census tract. Associations with race and ethnicity were modified by previous infraction-only arrests.

**Conclusions:**

This study confirms that many previously studied correlates of firearm recovery are still relevant today. We were able to expand on previous research by examining novel associations including purchasers’ criminal history and previous firearm transaction history. These results provide evidence that can be used to disrupt firearm use in crimes.

**Supplementary Information:**

The online version contains supplementary material available at 10.1186/s40621-024-00491-8.

## Background

Firearm violence is a critical public health problem in the United States (US) (American Medical Association 1989). From 2010 to 2020, interpersonal firearm violence accounted for an estimated 395,177 deaths and 1,156,594 non-fatal injuries in the US (Centers for Disease Control and Prevention 2002). Our understanding of how firearms move from legal purchase to criminal use is outdated and remains incomplete. Identifying predictors of firearms used in crimes can improve our understanding of illegal firearm possession, help prevent firearm deaths and injuries, and increase neighborhood safety – an often-overlooked consequence of firearm violence that has multiple downstream effects on the population’s health across the lifespan (Carter et al. 2023; DePriest et al. 2018; Won et al. 2016).

Past studies conducted using firearm trace data from over 20 years ago (Wintemute 2009; Wintemute et al. 2004; Wright et al. 2010; Koper 2013; Wintemute et al. 2005; Koper 2007; Pierce et al. 2003; Pierce et al. 2006) identified a broad set of risk factors associated with the recovery of a firearm in a crime or with a shorter time to crime, including characteristics of firearm dealers, handguns, and purchasers. For firearm dealers, being located in a city center (Wintemute 2009; Koper 2013; Koper 2007), prior sales of firearms that became crime guns (Koper 2013; Koper 2007), and the percent of sales that were denials (Wintemute 2009; Wintemute et al. 2005) were positively associated with a firearm being recovered in a crime. In contrast, a retailer’s selling more firearms to police was protective (Wintemute et al. 2005). Firearms that were semi-automatic, medium or larger caliber handguns, easily concealable, and inexpensive, and had a higher ammunition capacity were more likely to be recovered in crimes (Wright et al. 2010; Koper 2013; Wintemute et al. 2005; Koper 2007; Pierce et al. 2003). Firearm purchaser characteristics positively associated with that firearm’s recovery in a crime included being younger (Wright et al. 2010; Koper 2013; Wintemute et al. 2005; Koper 2007), being female (Wright et al. 2010; Koper 2013; Koper 2007), being of Black vs white race (Koper 2013; Koper 2007), having a prior misdemeanor conviction (Wintemute et al. 2001; Wintemute et al. 1998), and making multiple purchases within a short time period (Wintemute et al. 2004; Wright et al. 2010). Identification of such predictors of firearms used in crimes at the dealer, firearm, and purchaser level allows for the design of multiple specific interventions to reduce firearm injury and death. For instance, in several states, there are now restrictions on how many handguns can be purchased within a 30-day period and bans on firearms that do not meet certain design and safety standards (Koper 2005; Lee et al. 2017; Webster et al. 2002; Cal. Code Regs. 2001; Penal Code and §27535 2022; Rev Stat and § 2019,pp. 58-3 2019; Code of Virginia 2006).

More recent studies using firearms trace data examine predictors of crime guns in select cities (Braga et al. 2022; Braga et al. 2021; Ciomek et al. 2020; Hureau and Braga 2018). In New York City and Boston, studies have used firearm trace data alongside in-depth qualitative interviews to understand ways in which prohibited persons acquire firearms (Braga et al. 2021; Hureau and Braga 2018). Firearms recovered in Boston (Hureau and Braga 2018) and New York (Braga et al. 2021) from high risk individuals or high crime neighborhoods, respectively, were more likely to be a handgun (Braga et al. 2021; Hureau and Braga 2018), have a low-quality manufacturer (Braga et al. 2021), had a longer time-to-crime (Hureau and Braga 2018), were more likely to be originally purchased in a southern state (Braga et al. 2021; Hureau and Braga 2018), and the original purchaser was not the firearm possessor the vast majority of the time (> 90%) (Braga et al. 2021; Hureau and Braga 2018). Firearms recovered in Boston that were associated with a network of co-offending individuals were more likely to be handguns, have illegal possession circumstances, and to have changed ownership at least once than firearms not associated with this network (Ciomek et al. 2020). In Oakland, firearms recovered in violent crimes as compared to other crimes were more likely to be privately manufactured firearms, recovered in the COVID-19 pandemic years as opposed to prior, and to have popular semiautomatic pistol calibers (Braga et al. 2022). These more recent studies focus on preventing firearm injuries in high-risk individuals, neighborhoods, or timeframes.

The present study expands on past research by following firearms sold statewide from the first point of sale through subsequent transactions to recovery in a crime to examine a broader range of potential correlates of firearm recovery in crimes. Our aims and hypotheses were the following:

1. To revisit previously documented associations between dealer, firearm, and purchaser characteristics and firearm recovery in crimes and to examine potential predictors of crime gun recovery that have not previously been studied, including the criminal history of purchasers and reports that a firearm was lost or stolen. Though we hypothesize that many firearm characteristics associated with recovery in crimes will remain the same, changes over the past 20 years in policing patterns, highly publicized crackdowns on firearm dealers, increased availability of firearms, and new legislation may have altered those associations.
2. To identify whether purchaser firearm possession modifies the association between purchaser characteristics and firearm recovery in crimes. While we do not have information about possessor at the time of recovery, we are able to identify firearms that we know to be no longer in the possession of the last purchaser. We hypothesize that purchaser characteristics would not be associated with firearm recovery when the purchaser was known to be no longer in possession of the firearm.
3. To assess whether the associations of dealer, firearm, and purchaser characteristics differ among handguns recovered in violent crimes versus weapons offenses. We are interested in examining these crime types separately given the well-documented racial disparities in low-level discretionary offenses such as weapon possession, which may result in an overreporting of weapons offenses among people of color, and the distinct and direct impact that violent crimes have on public health and individuals’ sense of safety in their neighborhood. As such, we hypothesize that associations between purchaser race and ethnicity and firearm recovery might differ between firearms recovered in violent and weapons crimes.
4. To evaluate whether associations differ among handguns recovered within a short time-to-crime by examining characteristics associated with handgun recovery within 1 and 3 years of the most recent purchase. A short “time-to-crime”, often defined as recovery within 1 or 3 years of purchase, is thought to be an indicator of potential weapons trafficking (Koper 2007; Pierce et al. 2003). We hypothesize that associations found in our first and second analyses would be stronger when examining the subset of firearms with a short time-to-crime.
5. To conduct an exploratory evaluation of racial disparities in handgun recovery in crimes by assessing how associations between purchaser race and ethnicity and firearm recovery are related to institutional, interpersonal, or structural processes that create and perpetuate racial disparities. To do this, we explore two interactions with race and ethnicity. First, we examine whether associations varied by neighborhood racial and ethnic composition, as majority non-white neighborhoods can be both over- and under-policed and this may impact the type and number of firearms recovered in crimes. Second, we assess whether prior arrest for an infraction without any other criminal charge modified the association of race and ethnicity. Infractions are low-level offenses that involve a high degree of police discretion and reflect socioracial disparities in policing practices. Prior infraction arrest without any other charge may provide a proxy for discretionary policing of low-level offenses that have been shown to be racially disparate relative to the underlying behavior.
6. To examine correlates of long guns recovered in crime. To our knowledge, no study has examined these correlates. As with prior research, our primary interest was in handguns given the vast majority of firearms used in crimes are handguns (Wright et al. 2010) (over 70% of crime guns in our data). We hypothesize that associations with dealer and purchaser characteristics would be similar but that firearm characteristics, including past transactions, would differ.

To evaluate these aims, we analyze 4,288,741 handguns and 2,665,669 long guns legally purchased in California between 1996 and 2020, 45,324 of which were recovered in crimes from 2010–2021, using statewide data on crime gun recovery records, Dealer Records of Sales (DROS records), and firearm purchaser criminal history.

## Methods

### Data

The main sources of data for this study were California crime gun recovery records from 2010–2021, California’s DROS records from 1996–2021, and firearm purchaser criminal history records from 1981–2021. The crime gun and DROS records are maintained in the California Department of Justice (CA DOJ) Automated Firearms System (AFS); criminal history data are maintained in CA DOJ Automated Criminal History System (ACHS).

Since the early 2000s, California has required that all firearms recovered by law enforcement be submitted to CA DOJ for the purpose of tracing through the Bureau of Alcohol, Tobacco, Firearms and Explosives (ATF) and that CA DOJ maintain the records for at least ten years. These gun trace records include details on the firearm (make, model, caliber or gauge, and serial number) as well as the type and date of transaction, including purchases, pawns or pawn redemptions (when an individual recovers their firearm from a pawnshop by paying off their loan), firearms recovered by law enforcement agencies (LEAs), reports that a firearm is stolen, lost, or no longer in possession, any transfers of ownership, and new registrations. California is one of 15 states that require private citizens to report when a firearm is lost or stolen to a law enforcement agency (Firearms and Explosives 2023), and, since July 2017, this report must be filed within five days of the time the owner discovered or reasonably should have discovered the loss or theft (Penal Code and § 25265 2022). Also maintained by CA DOJ are crime gun recovery records, which are made by LEAs and include information on the date and type of crime in which the firearm was used.

The firearm trace records were linked to DROS records using CA DOJ linkage number (OCA and Date of Transaction). California law requires that transfers of firearms in the state be done through a licensed firearms retailer, including private party transfers. Thus, DROS records contain all legal handgun transactions since 1996 and transactions for rifles and shotguns since 2014 in California. This includes transfers between private parties, gun show sales, gifts, loans, and redemptions of pawned or consigned weapons. Prospective firearm purchasers must submit an application to the licensed retailer, who provides purchaser information to CA DOJ through electronic transfer. CA DOJ checks available state and federal records to determine whether the applicant is legally disqualified from purchasing or possessing firearms under state or federal laws. The DROS records contain information on the firearm; prospective purchaser (name, date of birth, sex, race and ethnicity, citizenship, country of origin, and address); the date, time, and type of transaction (e.g., sale, denial, transfer, pawn, etc.); Firearm Safety Certificate Exemption Codes (e.g., for police officers and active-duty military), and identifiers for the seller.

Firearm purchaser criminal history records were then linked on purchaser identification number provided by CA DOJ and date, to ensure that only criminal history prior to the firearm purchase was included. These records contain information on offense code, disposition, arrest date, conviction date, and level of crime (i.e., infraction, misdemeanor, or felony), and additional modifiers and comments. Crime type was defined using categories defined by the Uniform Crime Reporting Handbook using the offense information provided.

In total, our dataset contains 5,247,348 handgun and 2,868,713 long gun transaction records for 4,288,741 unique handguns and 2,665,669 unique long guns from 1996 to 2021. Firearms were included from their first DROS transaction until their recovery in a crime, their destruction by a law enforcement agency, or the end of administrative follow-up in 2021, whichever was sooner. Firearms that were only in California’s gun trace records as transaction types reported by LEAs (i.e., stolen, held for evidence, found, lost, held for safekeeping, a crime gun, having an institutional registration, under observation, retained for official use, or destroyed) and that did not link to any DROS records were excluded from the dataset as we would not have been able to identify any dealer, purchaser, or firearm characteristics supplied by the DROS records or link to purchaser criminal history (n = 31,403 handguns and 11,149 long guns). Transactions involving firearms that were destroyed or recovered in a crime prior to the study period or from firearm-purchaser pairs that concluded prior to the study period (e.g., the firearm was sold to another purchaser) were similarly excluded. A description of firearms and firearm transactions included vs. excluded in the final analytic dataset is depicted in Additional file 1: Figure S1.

This project was approved by the UC Davis Institutional Review Board.

### Measurements

#### Outcome measurements

Firearms recovered in crimes were identified using California crime gun recovery records. Time to firearm recovery in a crime was calculated as the time elapsed from the date a specific purchaser first purchased a firearm to the date of its recovery. Type of crime was classified as weapons or violent crime indicated by CA DOJ uniform offense code crime categories. Violent crimes included homicide (09xx), kidnapping (10xx), sexual assault (11xx), robbery (12xx), assault (13xx), and threats (16xx). Suicides were excluded from violent crime (codes 0914, 0915). Weapons offenses (52xx) included, among other things, carrying a concealed or prohibited weapon; possession, firing, or selling a weapon; and weapon trafficking.

### Covariate measurements

#### Characteristics of the dealer

Average sales, percent denials, percent of sales that were pawns or pawn redemptions, percent of guns sold in the calendar year that became crime guns that year, and percent of sales to police in the previous calendar year were calculated from DROS and firearm recovery records. These variables were lagged 1 year. The percent of sales to police was categorized as ≤ 5%, 5 to ≤ 10%, 10 to ≤ 20%, and > 20%. Dealerships which were not open in the previous calendar year were categorized as missing.

#### Characteristics of the firearm

Median manufacture firearm prices were compiled from the Blue Book of Gun Values (2017). We used the Possibly Gapped Histogram procedure (Fushing and Roy 2018) to identify low-cost manufactures as a proxy for inexpensive handguns. Whether a firearm was semiautomatic was determined from DROS records. For handguns, caliber was categorized as small (e.g., 0.22, 0.25, or 0.32), medium (e.g., 0.38 or 9 mm), or large (e.g., 0.40, 0.44, or 0.45) according to the firearm caliber, make, and model provided by DROS records. Long guns were classified as rimfire rifles, centerfire rifles, frame/receiver only rifles, and shotguns excluding 410-shotguns using information on firearm type and caliber from DROS records. Other calibers of firearms were excluded from analysis as they were uncommon, for handguns this included frame/receiver only and interchangeable barrel firearms (n = 59,814 handguns, 1.33% of handguns), and for long guns this included frame/receiver only, interchangeable barrel, 410 shotguns, and rifle/shotgun combinations (n = 70,777 long guns, 2.59% of long guns). Information on the firearms’ previous transactions (law enforcement holds, consignments, intrafamilial transfers, law enforcement release, being lost, pawn redemptions, pawns, and thefts) were determined from AFS firearm trace records and DROS.

#### Characteristics of the purchaser

The age, sex, citizenship status, place of birth (in the US or not), and race and ethnicity of the last recorded purchaser were determined from DROS records. The number of handguns purchased in the previous year, being a first-time handgun purchaser, and possessing only long guns were calculated in a time-varying manner from DROS records. Dealer and purchaser addresses were geocoded and distance between the purchaser and dealer was calculated. Dealer addresses with unmappable locations (e.g., post office boxes) were set to missing (10.6% of dealer addressees). Distance from purchaser address to dealership was categorized as missing, ≤ 5 miles, 5 to ≤ 20 miles, and > 20 miles.

From purchaser criminal history, we included indicators for any arrests within the past ten years for alcohol intoxication, firearm-related crimes, major violent crime, and major property crime. An arrest for an infraction without other charges within ten years prior to firearm purchase was included as an indication of potential over-policing of an individual.

The social vulnerability index (SVI) subscales (socioeconomic status, racial and ethnic minority status, household characteristics, and housing type and transportation), and 2010 Rural–Urban Commuting Area (RUCA) codes of the purchaser’s census tract were also included. The SVI score indicates the relative vulnerability of a census tract to hazardous events with a higher number indicating more vulnerability. SVI and its subscales were chosen as a measurement of neighborhood characteristics as they were available for all study years at the census tract level and have previously been related to neighborhood crime (Polcari et al. 2022). RUCA code was dichotomized as 1 (most urban) vs other.

#### Characteristics of the purchaser-firearm pair

A time-varying indicator of whether the firearm was lost, stolen, found, or no longer in possession was also included to indicate whether the last recorded purchaser was no longer in possession of the firearm.

### Statistical analysis

We defined our study period as 2010–2021. California’s crime gun law only requires that records for recovered crime guns be maintained by CA DOJ for 10 years, thus data on crime guns is more robust from 2010 onwards (Penal Code and §11108  2017). As such, we excluded handguns that had been destroyed or recovered in a crime prior to January 1, 2010 (n = 12,667 handguns) to capture only guns in circulation that were at risk of becoming a first-time crime gun. Similarly, reporting for long gun transactions was not required until 2014, so we excluded long guns destroyed or recovered in a crime prior to January 1, 2014 (n = 45). In total, 4,288,741 handguns and 2,665,669 long guns were included in analyses. To assess whether firearms excluded from the analyses differed from firearms included in the analyses, we compared the distributions of firearm and transaction covariates.

Analysis for handguns and long guns was conducted separately since correlates of recovery in crime may differ. Second, the reporting of handguns and long guns in DROS has been required for differing amounts of time. For all analyses pertaining to handguns, we examined associations among all crime guns and then separately among firearms recovered in weapons crimes and violent crimes.

We first compared the distributions of covariates among firearms that were and were not recovered in a crime using mean and standard deviations (SD) for continuous covariates and n (%) for categorical and dichotomous covariates.

We then assessed which characteristics were robust to multivariable adjustment by including all covariates in a Cox proportional hazard model. Firearm transaction was the unit of analysis. Models accounted for left truncation (time since initial purchase by the current purchaser to 2010 for handguns or to 2014 for long guns) by specifying the date of first purchase by the current purchaser as the entry time in PROC PHREG in SAS. For all models, we included interaction terms for each characteristic of the purchaser and whether the last purchaser was known to be not in possession of the firearm because the firearm had been reported lost, stolen, or no longer in possession. This was done as we do not have information on possessor of the firearm at the time that the firearm was recovered by law enforcement, but can infer whether the last known purchaser was not in possession of the firearm when it was recovered in a crime (i.e., firearm was reported stolen, lost, or not in possession). All models also accounted for clustering between transactions of the same firearm-purchaser pair, the firearm, the purchaser, the dealership the firearm was purchased at, and the purchaser’s and dealership’s census tract.

Only covariates that remained significant at *P* < 0.05 were retained in the final model and are presented in Table 1 and Additional file 1: Table S4. As P-values directly relate to sample size and our sample size is over 2 million both for handguns and long guns, we also present the chi-square value in tables with multivariable adjusted covariates to allow for an assessment of the strength of the relative associations.

**Table 1:** Multivariable adjusted characteristics associated with a handgun being picked up in a crime

Characteristic	All crime guns			Handguns picked up in violent crimes			Handguns picked up in weapons crimes		
	Hazard Ratio (95% CI^1^)	χ-Square	P-value	Hazard Ratio (95% CI)	χ-Square	P-value	Hazard Ratio (95% CI)	χ-Square	P-value
**Characteristics supplied by dealer**									
Average sales per year, per 1000	1.01 (1.01, 1.02)	11.9	0.001	1.04 (1.02, 1.06)	17.7	< 0.0001	–		
Average percent denials	1.04 (1.04, 1.05)	214.2	< 0.0001	1.05 (1.04, 1.06)	140.2	< 0.0001	1.04 (1.03, 1.05)	105.4	< 0.0001
Average percent of handgun sales that are pawn redemptions	1.01 (1.00, 1.01)	8.0	0.005	1.02 (1.01, 1.03)	23.7	< 0.0001	–		
Average percent of handgun sales that are pawns	1.01 (1.00, 1.01)	14.5	0.0001	–			1.01 (1.01, 1.02)	50.3	< 0.0001
Average percent of sales in calendar year that become crime guns that year	1.22 (1.16, 1.28)	55.6	< 0.0001	1.20 (1.14, 1.26)	45.2	< 0.0001	1.22 (1.16, 1.28)	57.6	< 0.0001
***Percent sales to police in the past year***									
Missing (dealership not open)	0.97 (0.91, 1.04)	0.7	0.41	1.01 (0.83, 1.23)	0	0.93	0.93 (0.85, 1.00)	3.6	0.06
≤ 5%	1.00 (0.96, 1.03)	0.1	0.79	1.08 (0.98, 1.19)	2.4	0.12	0.95 (0.91, 0.99)	6.6	0.01
> 5–10%	1.10 (1.07, 1.13)	43.2	< 0.0001	1.06 (0.97, 1.16)	1.6	0.20	1.07 (1.04, 1.11)	16.1	< 0.0001
> 10–20%	Reference			Reference			Reference		
> 20%	0.79 (0.76, 0.83)	106.8	< 0.0001	0.78 (0.68, 0.9)	12.4	0.0004	0.75 (0.71, 0.79)	106.4	< 0.0001
**Characteristics of the firearm**									
Low-cost manufacturers	1.71 (1.63, 1.79)	512.2	< 0.0001	1.73 (1.50, 1.99)	57.5	< 0.0001	1.76 (1.66, 1.86)	376.6	< 0.0001
Semiautomatic	1.17 (1.13, 1.21)	93.9	< 0.0001	1.26 (1.14, 1.39)	19.1	< 0.0001	1.21 (1.16, 1.26)	86.1	< 0.0001
***Caliber size***									
Small	Reference			Reference			Reference		
Medium	1.22 (1.17, 1.27)	88.4	< 0.0001	1.40 (1.22, 1.60)	23.5	< 0.0001	1.13 (1.08, 1.19)	23.4	< 0.0001
Large	1.28 (1.23, 1.34)	127.7	< 0.0001	1.37 (1.19, 1.58)	19.5	< 0.0001	1.23 (1.17, 1.30)	61.4	< 0.0001
Any previous law enforcement holds	0.55 (0.52, 0.59)	293.9	< 0.0001	0.61 (0.50, 0.75)	23.0	< 0.0001	0.46 (0.42, 0.50)	308.1	< 0.0001
Any previous intrafamilial transfer	0.66 (0.50, 0.88)	7.8	0.005	–			–		
Any previous law enforcement release	1.16 (1.07, 1.27)	11.4	0.001	–			1.27 (1.14, 1.41)	18.9	< 0.0001
Any previous lost	2.99 (2.54, 3.52)	174.2	< 0.0001	3.41 (2.02, 5.76)	21.1	< 0.0001	3.03 (2.51, 3.65)	135.2	< 0.0001
Any previous pawn redemption	1.15 (1.01, 1.31)	4.7	0.03	–			0.58 (0.51, 0.66)	68.2	< 0.0001
Any previous pawn	0.56 (0.49, 0.63)	77.9	< 0.0001	0.64 (0.47, 0.86)	8.5	0.0035	–		
Any previous stolen	8.93 (7.72, 10.34)	862.0	< 0.0001	9.53 (5.70, 15.95)	73.6	< 0.0001	8.56 (7.22, 10.14)	616.5	< 0.0001
**Characteristics of the purchaser**									
***In possession of handgun***									
Age, per 10 years	0.71 (0.70, 0.72)	3628	< 0.0001	0.72 (0.70, 0.74)	365.3	< 0.0001	0.68 (0.67, 0.69)	2710	< 0.0001
Female sex	1.37 (1.32, 1.41)	338.6	< 0.0001	1.37 (1.24, 1.51)	38.5	< 0.0001	1.41 (1.35, 1.47)	263.1	< 0.0001
Citizen status	0.68 (0.63, 0.73)	105.1	< 0.0001	0.67 (0.55, 0.81)	17.4	< 0.0001	0.68 (0.62, 0.75)	62.4	< 0.0001
Foreign born	0.95 (0.91, 0.99)	7.0	0.01	–			0.90 (0.86, 0.95)	15.4	< 0.0001
***Race and ethnicity***									
Asian	0.83 (0.79, 0.87)	53.2	< 0.0001	0.95 (0.82, 1.09)	0.6	0.44	0.82 (0.76, 0.87)	38.0	< 0.0001
Black	3.72 (3.59, 3.85)	5452	< 0.0001	4.24 (3.82, 4.70)	731.1	< 0.0001	4.06 (3.89, 4.24)	4143	< 0.0001
Hispanic	1.43 (1.38, 1.47)	513.6	< 0.0001	1.51 (1.37, 1.66)	73.6	< 0.0001	1.43 (1.38, 1.49)	337.2	< 0.0001
Native American/Pacific Islander	1.52 (1.40, 1.64)	103.4	< 0.0001	1.44 (1.11, 1.87)	7.8	0.005	1.68 (1.52, 1.85)	110.6	< 0.0001
Other	1.51 (1.38, 1.67)	72.3	< 0.0001	1.85 (1.41, 2.42)	19.9	< 0.0001	1.52 (1.34, 1.71)	45.4	< 0.0001
White	Reference			Reference			Reference		
***Handguns bought in the last year***									
0	Reference			Reference			Reference		
1	0.95 (0.91, 1.00)	4.5	0.03	0.85 (0.74, 0.98)	4.7	0.03	0.94 (0.89, 0.99)	4.7	0.03
2–5	0.73 (0.69, 0.77)	122.4	< 0.0001	0.75 (0.63, 0.89)	10.6	0.001	0.71 (0.66, 0.76)	91.0	< 0.0001
6–12	0.70 (0.62, 0.78)	38.5	< 0.0001	0.71 (0.49, 1.01)	3.7	0.06	0.76 (0.66, 0.87)	15.4	< 0.0001
> 12	1.60 (1.42, 1.80)	60.0	< 0.0001	0.27 (0.11, 0.66)	8.3	0.004	2.38 (2.10, 2.70)	183.2	< 0.0001
First handgun purchase	1.34 (1.30, 1.38)	359.0	< 0.0001	1.50 (1.37, 1.65)	73.7	< 0.0001	1.27 (1.23, 1.32)	161.7	< 0.0001
***Criminal history, arrests***									
Infraction with no other charge, past 10 years	2.28 (2.03, 2.57)	183.8	< 0.0001	2.68 (1.92, 3.75)	33.6	< 0.0001	2.47 (2.15, 2.84)	160.6	< 0.0001
Alcohol intoxication, past 10 years	1.88 (1.81, 1.95)	1118	< 0.0001	1.84 (1.64, 2.06)	112.1	< 0.0001	1.86 (1.78, 1.95)	697.5	< 0.0001
Firearm-related, past 10 years	2.07 (1.92, 2.24)	339.6	< 0.0001	1.85 (1.46, 2.35)	26.0	< 0.0001	2.08 (1.89, 2.28)	231	< 0.0001
Major violent crime, past 10 years	2.10 (1.95, 2.25)	436.7	< 0.0001	2.49 (2.05, 3.03)	83.8	< 0.0001	2.13 (1.96, 2.32)	307.6	< 0.0001
Major property crime, past 10 years	2.36 (2.19, 2.54)	513.3	< 0.0001	2.27 (1.81, 2.84)	50.7	< 0.0001	2.49 (2.28, 2.72)	410.8	< 0.0001
***Distance to dealer***									
Missing	0.94 (0.90, 0.99)	5.1	0.02	1.11 (0.96, 1.28)	1.9	0.17	0.92 (0.86, 0.98)	7.3	0.007
≤ 5 miles	1.11 (1.08, 1.14)	64.3	< 0.0001	1.12 (1.04, 1.22)	8.2	0.004	1.12 (1.09, 1.16)	48.5	< 0.0001
5–20 miles	Reference			Reference			Reference		
> 20 miles	0.84 (0.81, 0.87)	106.9	< 0.0001	0.90 (0.82, 1.00)	3.9	0.047	0.84 (0.80, 0.87)	69.3	< 0.0001
***Purchaser geographic characteristics***									
SVI^1^, socioeconomic status, per 10 units	1.08 (1.07, 1.08)	329.6	< 0.0001	1.07 (1.05, 1.09)	39.7	< 0.0001	1.09 (1.08, 1.10)	279.9	< 0.0001
SVI, housing status, per 10 units	1.00 (0.99, 1.00)	0.1	0.7005	–			1.01 (1.00, 1.02)	10.9	0.001
SVI, racial and ethnic minority status, per 10 units	1.05 (1.04, 1.05)	163.6	< 0.0001	1.04 (1.02, 1.06)	15.0	0.0001	1.05 (1.04, 1.06)	113.7	< 0.0001
SVI, housing type and transportation, per 10 units	1.03 (1.02, 1.03)	115.8	< 0.0001	1.05 (1.03, 1.06)	33.5	< 0.0001	1.02 (1.01, 1.02)	32.3	< 0.0001
RUCA^1^ code 1	1.34 (1.29, 1.39)	202.2	< 0.0001	1.38 (1.22, 1.56)	25.4	< 0.0001	1.32 (1.26, 1.39)	122.0	< 0.0001
***Not in possession of handgun***									
Age, per 10 years	0.94 (0.93, 0.96)	29.3	< 0.0001	1.00 (0.93, 1.07)	0.0	0.91	0.93 (0.90, 0.95)	31.7	< 0.0001
Female sex	1.05 (0.98, 1.13)	1.8	0.18	0.94 (0.74, 1.21)	0.2	0.65	1.05 (0.96, 1.15)	1.0	0.31
Citizen status	0.93 (0.78, 1.10)	0.8	0.38	0.99 (0.60, 1.62)	0.0	0.95	0.89 (0.71, 1.10)	1.2	0.27
Foreign born	0.90 (0.82, 0.98)	5.5	0.02	–			0.87 (0.78, 0.98)	5.3	0.02
***Race and ethnicity***									
Asian	1.19 (1.07, 1.32)	10.9	0.001	1.66 (1.22, 2.25)	10.5	0.001	1.27 (1.11, 1.44)	12.8	0.0004
Black	1.19 (1.10, 1.29)	18.5	< 0.0001	1.37 (1.05, 1.79)	5.4	0.02	1.28 (1.16, 1.41)	23.4	< 0.0001
Hispanic	1.04 (0.97, 1.12)	1.3	0.25	1.26 (0.99, 1.59)	3.6	0.06	1.01 (0.92, 1.10)	0.0	0.87
Native American/Pacific Islander	1.05 (0.87, 1.27)	0.2	0.62	2.25 (1.38, 3.65)	10.7	0.001	1.00 (0.78, 1.28)	0.0	0.99
Other	1.05 (0.84, 1.31)	0.2	0.66	0.84 (0.35, 2.05)	0.1	0.71	1.16 (0.89, 1.52)	1.2	0.27
White	Reference			Reference			Reference		
***Handguns bought in the last year***									
0	Reference			Reference			Reference		
1	1.04 (0.96, 1.13)	0.8	0.36	1.01 (0.75, 1.38)	0.0	0.93	1.05 (0.94, 1.16)	0.7	0.40
2–5	0.94 (0.85, 1.05)	1.1	0.29	0.98 (0.68, 1.43)	0.0	0.93	0.89 (0.78, 1.02)	2.8	0.09
6–12	0.71 (0.52, 0.97)	4.7	0.03	0.44 (0.11, 1.79)	1.32	0.25	0.76 (0.52, 1.11)	2.0	0.16
> 12	0.65 (0.40, 1.05)	3.1	0.08	0.00 (0.00, 0.00)	3818	< 0.0001	0.59 (0.31, 1.09)	2.8	0.09
First handgun purchase	1.15 (1.08, 1.22)	19.1	< 0.0001	1.34 (1.09, 1.66)	7.42	0.01	1.13 (1.04, 1.22)	9.0	0.003
***Criminal history, arrests***									
Infraction with no other charge, past 10 years	0.94 (0.66, 1.34)	0.1	0.73	1.28 (0.47, 3.44)	0.2	0.63	0.88 (0.56, 1.40)	0.3	0.59
Alcohol intoxication, past 10 years	0.94 (0.86, 1.03)	1.7	0.19	0.79 (0.57, 1.09)	2.1	0.15	0.93 (0.83, 1.04)	1.6	0.20
Firearm-related, past 10 years	0.81 (0.68, 0.96)	5.6	0.02	0.40 (0.17, 0.90)	4.8	0.03	0.81 (0.65, 1.01)	3.7	0.06
Major violent crime, past 10 years	0.83 (0.71, 0.97)	5.7	0.02	0.75 (0.43, 1.32)	1.0	0.33	0.84 (0.69, 1.02)	3.1	0.08
Major property crime, past 10 years	0.94 (0.78, 1.14)	0.4	0.54	1.28 (0.72, 2.26)	0.7	0.40	0.87 (0.69, 1.11)	1.3	0.26
***Distance to dealer, km***									
Missing	1.01 (0.92, 1.10)	0.0	0.83	1.39 (1.04, 1.86)	5.0	0.03	0.97 (0.86, 1.08)	0.4	0.55
≤ 5 miles	1.02 (0.96, 1.09)	0.5	0.48	1.26 (1.03, 1.55)	4.8	0.03	1.03 (0.96, 1.11)	0.7	0.42
5–20 miles	Reference			Reference			Reference		
> 20 miles	0.95 (0.88, 1.02)	1.9	0.17	0.95 (0.73, 1.25)	0.1	0.73	0.98 (0.89, 1.08)	0.2	0.66
***Purchaser geographic characteristics***									
SVI, socioeconomic status, per 10 units	0.97 (0.95, 0.99)	13.9	0.0002	0.94 (0.89, 0.98)	7.3	0.007	0.96 (0.94, 0.98)	11.5	0.0007
SVI, housing status, per 10 units	0.99 (0.98, 1.00)	4.4	0.03	–			1.00 (0.98, 1.01)	0.4	0.54
SVI, racial and ethnic minority status, per 10 units	1.05 (1.03, 1.07)	39.5	< 0.0001	1.09 (1.04, 1.14)	12	0.0005	1.05 (1.03, 1.07)	22.6	< 0.0001
SVI, housing type and transportation, per 10 units	1.00 (0.99, 1.01)	0.3	0.60	1.03 (0.99, 1.07)	2.5	0.11	1.00 (0.98, 1.01)	0.1	0.77
RUCA code 1	1.19 (1.09, 1.31)	14.2	0.0002	1.05 (0.77, 1.44)	0.1	0.76	1.20 (1.07, 1.34)	9.1	0.003

All variables were entered into a Cox proportional hazard model which accounted for left truncation and clustering between transactions of the same firearm-purchaser pair, the firearm, the purchaser, the dealership the firearm was purchased at, and the purchaser’s and dealership’s census tracts

^1^Abbreviations: CI: Confidence Interval, SVI: Social Vulnerability Index, RUCA: Rural Urban Commuting Area

Following this analysis, we assessed associations of dealer, firearm, and purchaser characteristics with handgun recovery within 1 and 3 years of the most recent purchase. To do this, the analytic dataset was restricted to firearm transactions within 365 or 1095 days (1 or 3 years) of the most recent purchase. The last firearm transaction in this time frame used in analysis as this data point contained information on all past transactions within the time frame as well as the most recent purchaser and dealer characteristics. Covariates were entered into a Poisson regression model to estimate risk ratios and their 95% confidence intervals. Covariates that remained significant at the *P* < 0.05 were retained and are presented in Table 2.

**Table 2:** Multivariable adjusted characteristics associated with a handgun being picked up with a short time-to-crime

Characteristic	Crime guns recovered within 1 year			Crime guns recovered within 3 years		
	Risk Ratio (95% CI^1^)	t-Value	P-value	Risk Ratio (95% CI)	t-Value	P-value
**Characteristics supplied by dealer**						
Average sales per year, per 1000	0.98 (0.97, 0.99)	-6.4	< 0.0001	0.98 (0.97, 0.99)	-6.0	< 0.0001
Average percent denials	1.06 (1.05, 1.06)	29.3	< 0.0001	1.06 (1.06, 1.06)	29.9	< 0.0001
Average percent of handgun sales that are pawn redemptions	1.01 (1.01, 1.02)	7.0	< 0.0001	1.01 (1.01, 1.02)	7.6	< 0.0001
Average percent of sales in calendar year that become crime guns that year	1.21 (1.19, 1.23)	19.7	< 0.0001	1.21 (1.18, 1.23)	19.4	< 0.0001
***Percent sales to police in the past year***						
Missing (dealership not open)	1.20 (1.12, 1.29)	5.0	< 0.0001	1.20 (1.12, 1.28)	5.2	< 0.0001
≤ 5%	0.92 (0.89, 0.95)	-4.6	< 0.0001	0.92 (0.89, 0.95)	-5.0	< 0.0001
> 5–10%	1.08 (1.04, 1.11)	4.8	< 0.0001	1.06 (1.03, 1.09)	3.9	< 0.0001
> 10–20%	Reference			Reference		
> 20%	0.72 (0.69, 0.76)	-13.3	< 0.0001	0.73 (0.70, 0.77)	-13.2	< 0.0001
**Characteristics of the firearm**						
Low-cost manufacturers	2.12 (2.02, 2.22)	30.7	< 0.0001	2.03 (1.94, 2.12)	29.9	< 0.0001
Semiautomatic	1.12 (1.08, 1.16)	6.6	< 0.0001	1.13 (1.09, 1.16)	7.1	< 0.0001
***Caliber size***						
Small	Reference			Reference		
Medium	1.18 (1.13, 1.24)	7.4	< 0.0001	1.18 (1.13, 1.23)	7.5	< 0.0001
Large	1.42 (1.35, 1.48)	14.8	< 0.0001	1.40 (1.33, 1.46)	14.7	< 0.0001
Any previous law enforcement holds	0.70 (0.62, 0.79)	-5.8	< 0.0001	0.68 (0.63, 0.75)	-8.6	< 0.0001
Any previous law enforcement release	1.87 (1.60, 2.20)	7.7	< 0.0001	1.65 (1.48, 1.85)	9.0	< 0.0001
Any previous lost	4.42 (3.14, 6.22)	8.5	< 0.0001	3.68 (2.86, 4.73)	10.1	< 0.0001
Any previous pawn redemption	1.34 (1.18, 1.52)	4.4	< 0.0001	1.42 (1.27, 1.58)	6.4	< 0.0001
Any previous stolen	9.96 (7.25, 13.67)	14.2	< 0.0001	8.88 (6.99, 11.29)	17.9	< 0.0001
**Characteristics of the purchaser**						
***In possession of handgun***						
Age, per 10 years	0.70 (0.69, 0.70)	-66.9	< 0.0001	0.70 (0.69, 0.70)	-67.9	< 0.0001
Female sex	1.26 (1.22, 1.30)	-13.5	< 0.0001	1.25 (1.21, 1.3)	-13.5	< 0.0001
Citizen status	0.75 (0.70, 0.80)	-8.5	< 0.0001	0.75 (0.70, 0.8)	-8.6	< 0.0001
***Race and ethnicity***						
Asian	0.80 (0.76, 0.84)	-9.1	< 0.0001	0.79 (0.76, 0.83)	-9.5	< 0.0001
Black	3.15 (3.04, 3.26)	63.0	< 0.0001	3.18 (3.07, 3.29)	64.1	< 0.0001
Hispanic	1.29 (1.25, 1.33)	15.8	< 0.0001	1.28 (1.24, 1.32)	15.7	< 0.0001
Native American/Pacific Islander	1.43 (1.32, 1.55)	8.6	< 0.0001	1.44 (1.33, 1.56)	8.9	< 0.0001
Other	1.43 (1.30, 1.58)	7.3	< 0.0001	1.45 (1.32, 1.59)	7.6	< 0.0001
White	Reference			Reference		
***Handguns bought in the last year***						
0	Reference			Reference		
1	1.03 (0.99, 1.08)	1.4	0.18	1.04 (0.99, 1.09)	1.7	0.10
2–5	0.81 (0.77, 0.86)	-7.2	< 0.0001	0.81 (0.77, 0.86)	-7.1	< 0.0001
6–12	0.76 (0.68, 0.86)	-4.5	< 0.0001	0.78 (0.70, 0.88)	-4.2	< 0.0001
> 12	1.79 (1.59, 2.02)	9.6	< 0.0001	1.79 (1.59, 2.01)	9.6	< 0.0001
First handgun purchase	1.43 (1.39, 1.48)	23.3	< 0.0001	1.43 (1.39, 1.48)	23.6	< 0.0001
***Criminal history, arrests***						
Infraction with no other charge, past 10 years	2.24 (1.99, 2.52)	13.5	< 0.0001	2.27 (2.02, 2.55)	13.8	< 0.0001
Alcohol intoxication, past 10 years	1.91 (1.84, 1.98)	34.4	< 0.0001	1.92 (1.85, 1.99)	35.1	< 0.0001
Firearm-related, past 10 years	2.24 (2.08, 2.41)	21.4	< 0.0001	2.21 (2.06, 2.37)	21.6	< 0.0001
Major violent crime, past 10 years	2.20 (2.06, 2.35)	23.2	< 0.0001	2.23 (2.09, 2.38)	24.3	< 0.0001
Major property crime, past 10 years	2.41 (2.24, 2.59)	24.0	< 0.0001	2.45 (2.29, 2.63)	24.8	< 0.0001
***Distance to dealer***						
Missing	0.46 (0.43, 0.48)	-29.6	< 0.0001	0.45 (0.43, 0.48)	-30.1	< 0.0001
≤ 5 miles	1.09 (1.06, 1.12)	6.5	< 0.0001	1.09 (1.06, 1.12)	6.6	< 0.0001
5–20 miles	Reference			Reference		
> 20 miles	0.87 (0.84, 0.90)	-8.2	< 0.0001	0.87 (0.84, 0.90)	-8.3	< 0.0001
***Purchaser geographic characteristics***						
SVI^2^, socioeconomic status, per 10 units	1.06 (1.06, 1.07)	17.6	< 0.0001	1.06 (1.06, 1.07)	15.8	< 0.0001
SVI, housing status, per 10 units	–			1.00 (1.00, 1.01)	0.1	0.90
SVI, racial and ethnic minority status, per 10 units	1.05 (1.04, 1.06)	13.7	< 0.0001	1.05 (1.04, 1.05)	13.2	< 0.0001
SVI, housing type and transportation, per 10 units	1.03 (1.02, 1.03)	10.6	< 0.0001	1.03 (1.02, 1.03)	10.7	< 0.0001
RUCA^2^ code 1	1.36 (1.30, 1.42)	14.5	< 0.0001	1.36 (1.30, 1.41)	14.6	< 0.0001
***Not in possession of handgun***						
Age, per 10 years	1.04 (1.00, 1.07)	-1.9	0.05	1.05 (1.02, 1.07)	-3.6	0.0003
Female sex	1.08 (0.96, 1.21)	-1.3	0.20	1.04 (0.96, 1.13)	-1.0	0.31
Citizen status	0.91 (0.7, 1.17)	-0.7	0.46	1.06 (0.88, 1.27)	0.6	0.56
***Race and ethnicity***						
Asian	1.09 (0.91, 1.30)	0.9	0.35	1.18 (1.05, 1.33)	2.8	0.006
Black	1.05 (0.93, 1.19)	0.8	0.44	1.07 (0.98, 1.17)	1.4	0.15
Hispanic	0.96 (0.86, 1.09)	-0.6	0.56	0.99 (0.91, 1.08)	-0.2	0.81
Native American/Pacific Islander	0.96 (0.71, 1.30)	-0.3	0.79	1.01 (0.81, 1.26)	0.1	0.95
Other	1.30 (0.94, 1.80)	1.6	0.12	1.08 (0.84, 1.39)	0.6	0.54
White	Reference			Reference		
***Handguns bought in the last year***						
0	Reference			Reference		
1	1.02 (0.88, 1.19)	0.3	0.77	1.04 (0.94, 1.15)	0.7	0.50
2–5	0.96 (0.79, 1.16)	-0.4	0.69	0.91 (0.80, 1.03)	-1.5	0.13
6–12	0.74 (0.43, 1.29)	-1.1	0.29	0.75 (0.51, 1.09)	-1.5	0.13
> 12	0.30 (0.10, 0.94)	-2.1	0.04	0.47 (0.27, 0.81)	-2.7	0.01
First handgun purchase	1.01 (0.91, 1.12)	0.2	0.86	1.06 (0.99, 1.15)	1.6	0.11
***Criminal history, arrests***						
Infraction with no other charge, past 10 years	1.12 (0.70, 1.79)	0.5	0.63	1.00 (0.69, 1.45)	0.0	0.98
Alcohol intoxication, past 10 years	0.93 (0.81, 1.08)	-0.9	0.35	0.96 (0.87, 1.07)	-0.7	0.46
Firearm-related, past 10 years	0.83 (0.61, 1.11)	-1.3	0.21	0.80 (0.65, 1.00)	-2.0	0.05
Major violent crime, past 10 years	0.91 (0.70, 1.17)	-0.8	0.45	0.91 (0.75, 1.09)	-1.0	0.30
Major property crime, past 10 years	1.01 (0.79, 1.31)	0.1	0.92	0.97 (0.80, 1.19)	-0.3	0.80
***Distance to dealer, km***						
Missing	0.61 (0.51, 0.74)	-5.2	< 0.0001	0.62 (0.54, 0.71)	-6.7	< 0.0001
≤ 5 miles	0.97 (0.88, 1.08)	-0.5	0.60	0.97 (0.91, 1.04)	-0.8	0.41
5–20 miles	Reference			Reference		
> 20 miles	0.94 (0.83, 1.06)	-1.0	0.33	0.94 (0.86, 1.03)	-1.3	0.19
***Purchaser geographic characteristics***						
SVI, socioeconomic status, per 10 units	0.97 (0.95, 1.00)	-2.3	0.02	0.96 (0.94, 0.98)	-3.6	0.0003
SVI, housing status, per 10 units	–			0.99 (0.97, 1.00)	-2.0	0.04
SVI, racial and ethnic minority status, per 10 units	1.04 (1.01, 1.06)	2.9	0.004	1.03 (1.01, 1.05)	3.5	0.0005
SVI, housing type and transportation, per 10 units	0.99 (0.97, 1.01)	-1.0	0.33	1.00 (0.99, 1.02)	0.4	0.71
RUCA code 1	1.30 (1.11, 1.53)	3.2	0.001	1.24 (1.11, 1.39)	3.8	0.0002

^1^All variables were entered into a Poisson regression model

^2^Abbreviations: CI: Confidence Interval, SVI: Social Vulnerability Index, RUCA: Rural Urban Commuting Area

In supplemental analyses, to further interrogate racial disparities in firearm recovery, we assessed two interactions. First, we assessed whether neighborhood racial and ethnic minority status, measured by the SVI subscale, modified the association between a purchaser’s race and ethnicity and being in a vulnerable neighborhood (≥ 75th percentile vs < 75th percentile). We included an interaction term between the SVI racial and ethnic minority status subscale and purchaser race and ethnicity to assess the influence of neighborhood structural and institutional racism on the purchaser-crime gun association. Second, we additionally assessed whether a purchaser having an infraction-only arrest history within the 10 years prior to purchasing the handgun, evidence of policing of low-level behavior, modified the association in a similar manner. This analysis is considered exploratory as the number of arrests for an infraction-only crime is relatively small (n = 7,746). All analyses were conducted with SAS version 9.4 (SAS Institute Inc.).

## Results

In total, 38,462 handguns (0.80% of handguns) and 6,883 long guns (0.24% of long guns) with previous documented transactions in DROS were recovered in crimes from 2010 or 2014, respectively, to 2021. Among handguns, 3,935 were recovered in violent crimes and 24,441 were in weapons crimes. Median time-to-crime from the last known purchase was 2.07 years (Q1–Q3 0.79–4.22) for all handguns (1.91 years for violent crimes and 2.08 years for weapons crimes). For long guns, median time-to-crime was 1.80 years (IQR 0.82–3.06). Firearms excluded from the analysis were more likely to have been consigned, released from law enforcement, and pawned compared to firearms included in the analysis (Additional file 1: Table S1).

### Research Questions 1 and 2. Correlates of handguns recovered in all crimes

Notable differences in dealer, handgun, and purchaser characteristics are seen between handguns recovered in crimes and those that were not. The associations between purchaser characteristics and handgun recovery depend on whether the purchaser was known to be no longer in possession of the firearm (Additional file 1: Table S2).

#### Dealer characteristics

In the multivariable analysis, a dealer’s sales volume, the percent of a dealers’ prior transactions that were administrative denials, the percent of sales that were pawns or pawn redemptions in the previous calendar year, and the percent of sales in the past calendar year that became crime guns in the next calendar year were each positively related to a handgun becoming a crime gun. On the other hand, a dealership having over 20% (vs > 10–20%) of their sales to police in the past calendar year was inversely associated with a handgun becoming a crime gun (Table 1).

#### Firearm characteristics

Being an inexpensive handgun, proxied by low-cost manufacturer, was positively associated with a firearm being picked up in a crime (Hazard Ratio [HR] 1.71; 95% Confidence Interval [CI] 1.63, 1.79). Likewise, a handgun having a medium or a large caliber versus a small caliber was positively associated with becoming a crime gun. The hazard of becoming a crime gun for a firearm that had been previously lost or stolen was 2.99 (95% CI 2.54, 3.52) and 8.93 (95% CI 7.72, 10.34) times larger, respectively, than if the firearm had not been reported lost or stolen. A handgun having a previous pawn was inversely associated with firearm becoming a crime gun (Table 1).

#### Purchaser characteristics

Various demographic characteristics of the purchaser related to the handgun becoming a crime gun. These associations were attenuated or no longer significant when the last known purchaser was known to be no longer in possession. Age and the purchaser being a citizen were each inversely associated with the handgun being a crime gun, whereas female sex was positively related to this outcome. The purchaser being Black, Hispanic, Native American or Pacific Islander, and other race and ethnicity compared to White was positively related to a handgun recovery. The association was largest among Black purchasers (HR 3.72; 95% CI 3.59, 3.85). A purchaser having Asian race or ethnicity vs white race was inversely related to a handgun becoming a crime gun. (Table 1). These associations between race and ethnicity and crime gun recovery were attenuated when the last known purchaser was known to be no longer in possession of the firearm, with the exception of when a purchaser’s race or ethnicity was Asian (Table 1). The handgun being the first known handgun purchase by the purchaser was positively associated with that firearm being recovered in a crime (HR 1.34, 95% CI 1.30, 1.38).

Purchaser criminal history was also related to a handgun being recovered in a crime. A purchaser’s arrest for an infraction-only offense within the past 10 years prior to firearm purchase was associated with 2.28 (95% CI 2.03–2.57) times the hazard of a handgun being picked up in a crime. Likewise, an arrest for alcohol intoxication, a firearm-related crime, a major violent crime, or a major property crime within the past 10 years were each independently associated with a handgun being recovered in a crime (Table 1). When the purchaser was known to be no longer in possession of the handgun, criminal history was either not related to or inversely related to a handgun recovered in a crime (Table 1).

#### Purchaser geographic characteristics

Various traits associated with a purchaser’s geographic address were related to handgun recovery. Compared to a firearm dealer being > 5–20 miles from a purchaser’s address, a dealer being close (< 5 miles) was positively related to a handgun becoming a crime gun, whereas a dealer being farther away (> 20 miles) was inversely associated with this outcome. Purchaser’s census tract having a higher SVI for socioeconomic status, racial and ethnic minority status, and transportation were each related to a handgun becoming a crime gun. Likewise, the purchaser’s census tract being urban was associated with the handgun being picked up in a crime (Table 1).

### Research Question 3. Correlates of Handguns Recovered in Violent Crimes and Weapons Offenses.

#### Dealer characteristics

Sales volume was related to a handgun being recovered in a violent crime but not in weapons crimes. The percent of sales that were pawns was associated with handgun recovery in weapon offenses but not violent crimes. Other associations were similar among handguns recovered in weapon-related offenses and violent crimes (Table 1).

#### Firearm characteristics

The positive associations of being an inexpensive handgun and having medium or large caliber versus small caliber were stronger among firearms picked up in violent crimes than for those recovered in weapons crimes. These associations of being previously lost or stolen were similar among firearms recovered in violent and weapons crimes. Having a previous pawn was not associated with firearms picked up in weapons crimes (Table 1).

#### Purchaser characteristics

Associations of purchaser age, citizenship status, and sex did not vary much by crime type. Asian race or ethnicity vs white race was inversely associated with a firearm recovery in a weapons crime and not associated with firearms picked up in violent crimes. Contrarily, the association between a purchaser being Black race was larger for both violent crimes and weapons offenses. The handgun being the first known purchase was positively related to firearm recovery in both violent and weapons crimes. For violent crimes, purchasing 1 or more firearms in the past year versus 0 was inversely associated with a firearm being picked up in a violent crime. For weapon crimes, if a purchaser purchased > 12 versus 0 firearms in the past year, this was positively associated with firearm recovery in weapons crimes. Associations with purchaser criminal history were relatively consistent in analyses among handguns picked up in violent or weapons crimes (Table 1).

#### Purchaser geographic characteristics

There was little variability in these associations by recovery crime type (Table 1).

### Research Question 4. Correlates of handguns recovered within a short time-to-crime.

Covariates associated with handgun recovery in 1 or 3 years are largely similar to those of handguns recovered in any timeframe (Table 2). Some associations differ in magnitude. Inexpensive and large caliber handguns are more strongly positively associated with handgun recovery within 1 or 3 years. Likewise, a law enforcement release or a firearm being reported lost have stronger positive associations when recovered within 1 or 3 years. Finally, the association with the purchaser being female sex is weaker in firearms recovered within these shorter time frames.

### Research Question 5. Exploratory evaluation of racial disparities in handgun recovery in crimes

As a sensitivity analysis, we separately examined whether high racial or ethnic minority neighborhood composition, as measured by the SVI subscale, modified the association between race and ethnicity and crime gun recovery. Though associations were slightly higher in neighborhoods with low racial and ethnic minority composition, the general direction and magnitude of the associations between race and ethnicity and crime gun recovery were similar. This was consistent among firearms recovered in violent and weapons crimes (Additional file 1: Table S3).

We also examined whether a purchaser having a previous arrest in the past 10 years for an infraction but no other charges modified the association between race and ethnicity and crime gun recovery. For firearm purchasers of Black and Asian race or ethnicity versus white, having a previous infraction-only arrest significantly increased the risk of a firearm being picked up in a crime compared to those with no infraction history (HR_Black, has infraction_: 5.69; HR_Black, no infraction_: 3.70; HR_Asian, has infraction_: 1.76; HR_Black, no infraction_: 0.83, P-values-for-interactions < 0.05). The association of Asian race or ethnicity vs white among those with an infraction was stronger when examining handguns recovered in violent crimes (HR 3.93, 95% CI 1.12–13.82) whereas the association of Black race vs white was stronger when examining handguns picked up in weapons crimes (HR 7.22 95% CI 4.80–10.85) (Additional file 1: Table S4).

### Research Question 6. Correlates of long guns recovered in crimes

Dealer, firearm, and purchaser characteristics differed between long guns recovered in a crime and those never recovered (Additional file 1: Table S5). In the multivariable analysis, most associations were similar to those of handguns, although often attenuated (Additional file 1: Table S6). For instance, other than the association of female sex, associations of the purchaser’s demographic characteristics (age, citizenship status, race and ethnicity) and a long gun becoming a crime were smaller than those found in the analysis of handguns.

There were, however, several correlates of a long gun becoming a crime gun that differed from those of handguns. For instance, a previous intrafamilial transfer was positively associated with a long gun being picked up in a crime and inversely associated with handgun recovery in crime. Additionally, having 6 or more handgun purchases within the past year and only purchasing long guns were positively associated with a long gun becoming recovered in a crime. Rifles that were frame/receiver only vs center fire rifles were inversely associated with becoming a crime gun, whereas rim fire rifles and shotguns (which are not 410-shotguns) were more likely to become crime guns (Additional file 1: Table S6).

## Discussion

In this study of correlates of firearm recovery in crimes in California from 2010–2021, we confirmed many characteristics that had previously been documented in the literature using statewide data from the 1990s, and more recent data from select cities, are still relevant today. With some notable exceptions, these associations were consistent when examining predictors of handguns recovered in violent crimes or weapon offenses. Additional correlates were found, specifically related to the firearm’s transaction history, the purchaser’s citizenship and place of birth, and the purchaser’s criminal arrest history. Further examination of associations of purchaser race and ethnicity with firearm recovery in crime indicated that an infraction with no other charge in the past 10 years modified the association among Black and Asian firearm purchasers, increasing the risk that a handgun would be recovered in a crime. Finally, this was the first study to examine long gun recovery in crimes and we found many correlates of firearm recovery were similar to those of handguns.

Several previous studies examining predictors of firearms used in crimes have focused on characteristics of firearm dealerships (Wintemute 2009; Koper 2013; Wintemute et al. 2005; Koper 2007), as a disproportionate number of crime guns are sold at a small percentage of dealerships (Pierce et al. 2006; Braga et al. 2012). Similar to previous research, we found that larger sales volume (Wright et al. 2010; Koper 2007), the percent of sales denied (Wintemute 2009; Wright et al. 2010; Wintemute et al. 2005; Pierce et al. 2006), and the percent of prior sales that became crime guns (Koper 2013) at a firearm dealership were each positively associated with firearms becoming used in crimes, and, on the other hand, the percent sales to police (Wintemute et al. 2005) was inversely associated with this outcome. In prior research, a firearm being sold at a pawnshop was positively associated with firearm recovery, though this did not always remain significant in multivariable analysis (Wright et al. 2010; Koper 2013; Wintemute et al. 2005; Koper 2007). While we were not able to distinguish between pawnbrokers and other types of retailers, we instead examined the percent of dealers’ prior handgun sales that were pawns or pawn redemptions and found that these variables were each positively associated with firearm recovery in crimes. Notably, storefront address was missing for 10.6% of firearm retailers (10.5% of firearm transactions) in our sample, so we were not able to examine characteristics such as dealership urbanicity, which has previously been associated with a higher percentage of firearms being recovered in crimes (Wintemute 2009; Koper 2013; Koper 2007). Nevertheless, confirming that these characteristics of dealerships first examined 20 years ago are still relevant today can help inform law enforcement of potentially high-risk retailers, thus providing a strategy to interrupt the movement of firearms from legal purchase to illegal procurement and/or use in crimes.

Characteristics of firearms associated with recovery in crimes were remarkably consistent with previous research. Previous research using firearms trace data found that firearms that are inexpensive or made by a low-cost manufacturer (Wintemute 2009; Wright et al. 2010; Koper 2013; Koper 2007; Braga et al. 2021), semiautomatic (Wintemute et al. 2004; Koper 2013; Koper 2007), and a medium or large caliber (Wright et al. 2010; Koper 2013; Koper 2007) are more likely to be recovered in crimes. We found similar associations with these features and crime gun recovery.

While previous research using ATF trace data was only able to examine correlates of the first point of sale, we were able to trace the transaction history from first point of sale to the end of follow-up. Previous pawn redemptions and law enforcement release were each positively associated with a firearm being recovered later in a crime. Contrarily, previously pawning a firearm and a previous law enforcement hold were inversely associated with recovery in crimes, perhaps due to the time spent at the dealership or LEA, respectively. Findings suggest that when a gun is reported as lost or stolen, that gun is 2.99 or 8.93 times more likely to be subsequently used in a crime, respectively. The association with lost firearms is stronger when only examining handguns recovered within 1 or 3 years (4.42 and 3.68 times more likely), potentially indicating weapons trafficking following losing a handgun. Thus, considering measures to secure firearms from theft or loss should be a primary focus for prevention of firearm-related crimes moving forwards. The associations with lost or stolen guns are not unexpected (Koper 2007; Alper and Glaze 2019; Harlow 2001), though to our knowledge have not previously been examined in this manner as this data is not available in standard ATF trace data. As firearms can change hands multiple times, these additional features provide a more complete understanding of which firearms end up used in firearm-related crimes.

We were also able to examine which characteristics of the most recent purchaser were associated with crime gun recovery. Similar to prior studies, we found that the purchaser being a younger age (Wright et al. 2010; Koper 2013; Wintemute et al. 2005; Koper 2007; Pierce et al. 2006), female sex (Wright et al. 2010; Koper 2013; Koper 2007), living in a more urban neighborhood (Koper 2013; Koper 2007), and Black versus white race (Koper 2013; Koper 2007) were positively associated with firearm recovery in crimes. The purchaser being Hispanic, Native American or Pacific Islander, and other race and ethnicity versus white race were also positively associated with firearm recovery in crimes. Research suggests that a positive association of female sex with a firearm being recovered in a crime may be evidence of straw purchasing (Koper 2013; Koper 2007). These associations were all attenuated when the purchaser reported to be no longer in possession of the firearm. This attenuation was in line with our hypotheses for Research Question 2, as the purchaser was likely not the crime gun possessor.

To further interrogate the association between purchaser race and ethnicity and firearm recovery, we examined two interactions to assess racial disparities: neighborhood racial and ethnic minority status and a purchaser having a past infraction with no other charge, and we assessed if these interactions modified the associations. We included neighborhood racial composition as an indicator for structural or institutional racism and past infraction with no other arrests as a potential indicator of police deployment and arrest practices that disproportionately impact Black and Hispanic individuals. Though point estimates for Black, Hispanic, and Native American or Pacific Islanders were slightly lower for violent and weapons crimes when neighborhood racial and ethnic minority status was high, the estimates were broadly similar. For infractions only arrest history, on the other hand, the association between purchaser race and ethnicity and firearm recovery in crimes was modified. Specifically, for firearms recovered in violent crimes, there was a positive association between being Asian vs white when the purchaser had a past infraction and no association when they did not. For weapons crimes, the association between being Black vs white and a firearm being recovered in a weapons crime was higher when the purchaser had a past infraction versus when they did not. This may be evidence of “over-policing” of Black and Hispanic individuals who look threatening to law enforcement officials based on racial profiling or represent differential rates of police monitoring and stop and frisk practices as the effect was modified among weapons, but not violent, crime guns (White and Fradella 2016; Kirk 2008; Schleiden et al. 2020). These results should be considered exploratory and replicated in other studies.

Other purchaser characteristics that we assessed have, to our knowledge, not been directly examined in previous studies. These include the purchaser’s citizenship and birthplace, the number of handguns bought in the previous year, if it was the first purchase of a handgun, the distance between purchaser and dealer address, and the purchaser’s criminal history. A purchaser’s first handgun purchase and a shorter distance from the purchaser’s address to the dealer’s location were each positively associated with firearm recovery in weapon offenses and violent crime. A shorter distance from the purchaser’s address to the dealership may reflect an urban environment, though urbanicity was independently associated with firearm recovery, or it may indicate the importance of ease of access to firearms. Previous research has shown that off-premise alcohol outlet density and pawn firearms dealers are positively associated with increased levels of firearm assaults (Pear et al. 2023). The positive association that we find between first handgun purchase and crime gun recovery might reflect the fact that an individual who purchases a firearm, perpetrates and is subsequently convicted of a felony or prohibiting misdemeanor will no longer be allowed to legally purchase a firearm in the state. The mechanism explaining these associations deserves further investigation in subsequent studies if these findings are replicated.

Similarly, arrests for all five types of crimes examined were positively associated with firearm recovery in crime, regardless of crime type. A past misdemeanor offense has been associated with future criminality among firearm owners (Wintemute et al. 2001; Wintemute et al. 1998), however the direct link to firearm recovery in crimes is, to our knowledge, novel. Acquiring more than 12 handguns in the previous year (which is only legal in California for law enforcement officers, registered private party transfers, returns to owners, and certain other specific circumstances as the state limits purchasers to one firearm per thirty days (Penal Code and §27535 2022)), was inversely associated with handguns recovered in violent crimes. However, purchase of > 12 handguns in the past year related to 2.38 times more recovery in weapons crimes. The inverse association with violent crime could reflect purchases of firearms by law enforcement officials or other parties strictly following the California law. Koper et al. (2007) previously found that a firearm purchase within 30 days of another purchase was inversely associated with recovery of that firearm in a crime after Maryland instituted a similar one-firearm-per-month law to that of California and interpreted it as such. That same report found a positive association with buying multiple firearms within a short period of time and firearm recovery in crime prior to enactment of this law and interpreted buying multiple firearms prior to enactment of this law to be an indication of weapons trafficking (Koper 2007). Perhaps individuals intent on weapons trafficking, such as a recently sentenced former San Diego County Sheriff’s Captain (Press Release 2021), are acquiring weapons by using the exceptions in the law. This puzzling finding is a topic for further research.

Finally, this is the first study to examine associations of firearm recovery in crimes among long guns, which we examined in a separate secondary analysis. Long guns are not recovered in crimes as often as handguns (~ 25% of crime guns recovered are long guns in our data) and long gun sales have only been required to be reported in California since 2014. Many associations were similar to those of handguns. Long guns were 6.31 and 17.43 times more likely to be recovered in a crime when reported lost and stolen, respectively, again emphasizing the need to focus on securing firearms from loss or theft as a crime prevention strategy. As there were a small number of long guns recovered in crimes (n = 6,883), we were not able to examine differences between long guns recovered in weapons versus violent crimes.

Strengths of this study include our large sample size, coverage of the state of California (as compared to single cities) from 2010 to 2021, and the ability to trace firearms transactions from the first to last in-state sale to recovery in a crime. There are limitations as well. We did not have information on possessor of the firearm at the time of recovery in a crime. Although we attempted to address this in part by stratifying our analysis when the firearm was known to not be in the original purchaser’s possession (because it had been reported lost, stolen, or not in possession), this is likely only a fraction of firearms recovered from individuals who differed from the purchaser. Further, we could not examine associations between firearm possessor characteristics and firearm recovery. Second, this study is restricted to firearms legally purchased and recovered in California. ATF trace data indicates that, when a source state was identified, approximately one-third of firearms recovered in crimes in the state were purchased outside of California. This has varied over time, with ≤ 30% of firearms recovered from 2010–2015 being identified as purchased out-of-state, > 30–40% from 2016–2019, and 45% in 2020 (Laqueur et al. 2023). We are unable to make inferences on this subset of firearms. Additionally, we excluded 188,870 handguns and 211,235 long guns from our analyses because they did not link to DROS records – this included 4049 handguns and 3089 long guns were recovered in crimes. These firearms were more likely to have been consigned, pawned, and have a law enforcement release, which may have influenced our findings regarding these covariates. Our findings should not be extrapolated to firearms without a dealer record of sale in California, as these firearms differ with respect to caliber and transaction history. Fourth, as we excluded firearms recovered in suicide deaths, our findings do not extend to efforts to reduce firearm suicide.

Finally, while we find a positive association between purchaser race and ethnicity and firearm recovery, these analyses cannot disambiguate the extent to which this reflects racial disparities in surveillance practices and police behavior versus differential unlawful behavior. While studies comparing crime victimization surveys and self-reported offending data with violent crime arrests suggest that the racial gap in violent crime arrests is largely explained by greater involvement (likely due to poverty, historical structural racism, neighborhood deprivation, etc.) rather than differential detection (Skeem and Lowenkamp 2016), differential policing has been documented for discretionary crimes, particularly drug possession (Geller and Fagan 2010). Further, racial disparities in police stops, searches, and arrests are well documented (Kovera 2019; Kirk 2008; Schleiden et al. 2020; Austin and Allen 2000; Ousey and Lee 2008; Pierson et al. 2020). Our analyses indicating racial differences in the association between a previous infraction and weapon offense recovery may suggest that there is pattern of over-policing or the prevalence of other systemic mechanisms that perpetuate racial disparities in the justice system among Black individuals, and our findings should be interpreted within this context.

## Conclusions

We have expanded on previous work examining predictors of firearms recovered in crimes from 2010–2021. Results from prior work conducted in the 1990s and early 2000s have, for the most part, been replicated by this study. Several new associations have been found. These results, in combination with prior studies, provide evidence for strategies to interrupt firearm use in crimes. Reducing the number of firearms used in crimes can reduce injury and deaths, as well as make communities less violent.

### Supplementary Information

**Additional file 1. **Supplemental Figure and Tables.

## Data Availability

The data that support the findings of this study are not openly available due to reasons of sensitivity and legality. The data are maintained by the CA DOJ.

## Abbreviations

ACHS: Automated criminal history system
AFS: Automated firearms system
ATF: Bureau of alcohol, tobacco, firearms and explosives
CADOJ: California department of justice
CI: Confidence interval
DROS: Dealer records of sales
HR: Hazard ratio
LEA: Law enforcement agencies
RUCA: Rural–urban commuting area
SD: Standard deviation
SVI: Social vulnerability index
US: United States
