# Supplementary material for: Purchaser, firearm, and retailer characteristics associated with crime gun recovery: a longitudinal analysis of firearms sold in California from 1996 to 2021
Source: Inj Epidemiol. 2024 Feb 26;11:8. doi: 10.1186/s40621-024-00491-8 (PMC10898164; doi:10.1186/s40621-024-00491-8)
Supplement: Supplementary file 1 — Additional file 1. Supplemental Figure and Tables. [file 40621_2024_491_MOESM1_ESM.docx]

**Supplement to “*Purchaser, firearm, and retailer characteristics associated with crime gun recovery: A longitudinal analysis of firearms sold in California from 1996 to 2021*”**

## **Supplemental Figure 1.** Firearm and transaction flow diagram

## **
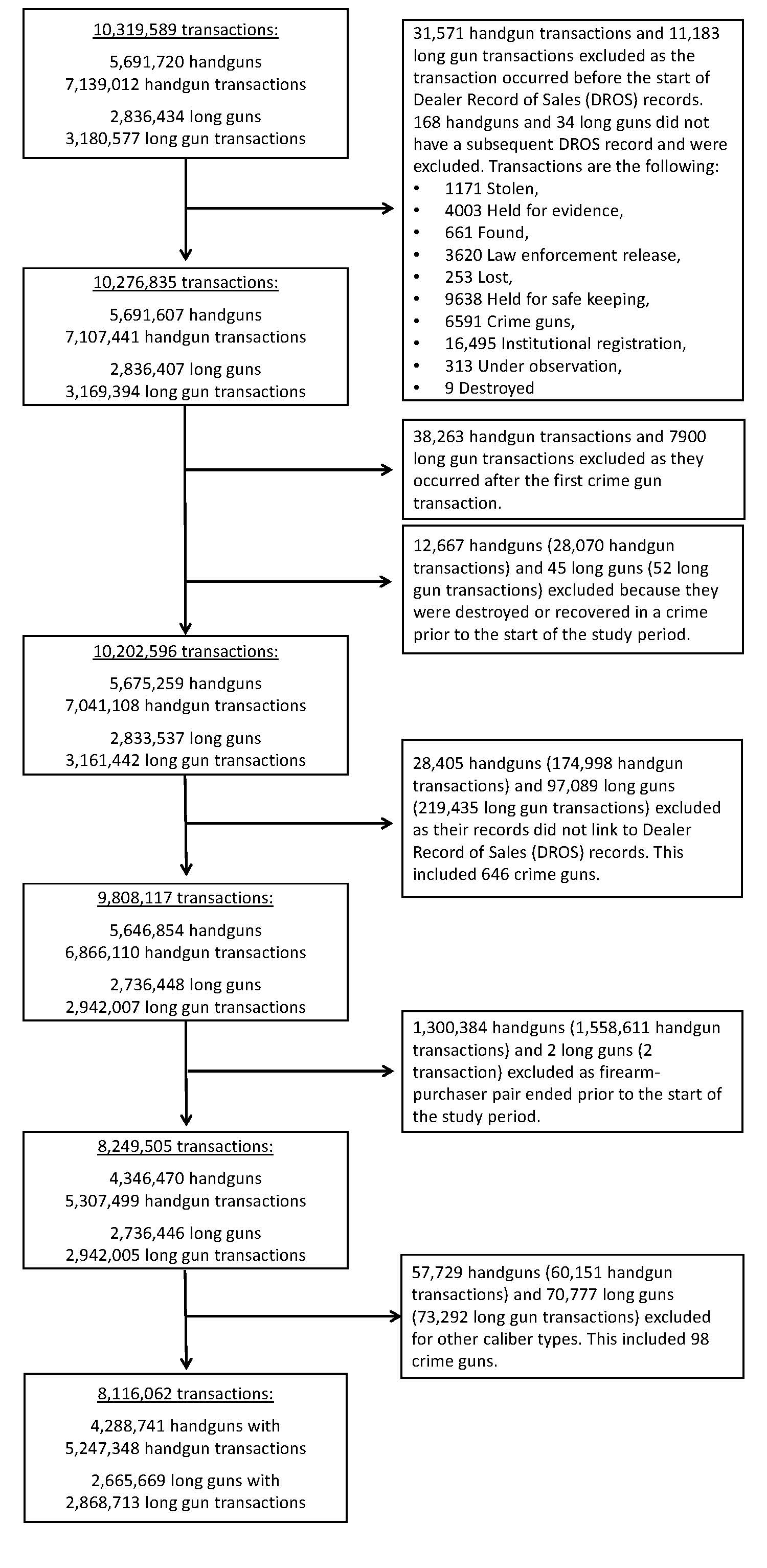
**

## **Supplemental Table 1.** Comparison of firearms and firearm transactions which linked and did not link to Dealer Record of Sales (DROS) records.

|  | **Handguns linked, n=4,288,741** | **Handguns not linked, n=188,870** | **Long guns linked, n=2,665,669** | **Long guns not linked, n=211,235** |
| --- | --- | --- | --- | --- |
| **Characteristic** | n (%) | n (%) | n (%) | n (%) |
|  |  |  |  |  |
| **Characteristics of the firearm** |  |  |  |  |
| Low-cost manufacturers | 95,852 (2.23) | 4767 (2.54) | -- | -- |
| Semiautomatic | 3,432,384 (81.14) | 115,362 (64.62) | 1,470,821 (55.18) | 111,070 (53.34) |
| Caliber size |  |  |  |  |
| Small | 466,013 (10.88) | 35,698 (19.03) | -- | -- |
| Medium | 2,207,483 (51.52) | 67,196 (35.82) | -- | -- |
| Large | 1,610,806 (37.60) | 84,717 (45.16) | -- | -- |
| Rifle, rimfire | -- | -- | 356,108 (13.36) | 77,827 (37.38) |
| Rifle, frame/receiver only | -- | -- | 451,188 (16.93) | 9728 (4.67) |
| Rifle, centerfire | -- | -- | 1,114,723 (41.82) | 70,655 (33.93) |
| Shotgun, not 410 | -- | -- | 743,650 (27.90) | 50,022 (24.02) |
| Any previous consignment | 58,257 (1.36) | 70,917 (37.75) | 11,445 (0.43) | 40,680 (19.54) |
| Any previous intrafamilial transfer | 14,045 (0.33) | 1614 (0.86) | 2978 (0.11) | 468 (0.22) |
| Any previous law enforcement release | 24,890 (0.58) | 2868 (1.53) | 4234 (0.16) | 1322 (0.63) |
| Any previous lost | 5166 (0.12) | 258 (0.14) | 372 (0.01) | 44 (0.02) |
| Any previous pawn redemption | 25,222 (0.59) | 187 (0.10) | 7027 (0.26) | 191 (0.09) |
| Any previous pawn | 20,983 (0.49) | 4816 (2.56) | 5859 (0.22) | 8062 (3.87) |
| Any previous stolen | 38,159 (0.89) | 1287 (0.69) | 4935 (0.19) | 350 (0.17) |
| Any crime gun | 38,441 (0.90) | 4049 (2.16) | 6806 (0.26) | 3089 (1.48) |

## **Supplemental Table 2**. Dealer, firearm, and purchaser characteristics by handgun recovery in a crime and crime type

|  | **Never a crime gun, n=4,250,300 firearms** | **All crime guns, n=38,441 firearms** | **Handguns picked up in violent crimes, n=3,935 firearms** | **Handguns picked up in weapons crimes, n=24,424 firearms** |
| --- | --- | --- | --- | --- |
| **Characteristic** | Mean (SD^1^)  or n (%) | Mean (SD)  or n (%) | Mean (SD)  or n (%) | Mean (SD)  or n (%) |
|  |  |  |  |  |
| **Characteristics supplied by dealer** |  |  |  |  |
| Average sales per year, per 1000 | 2.30 (1.84) | 2.39 (1.75) | 2.5 (1.82) | 2.36 (1.72) |
| Average percent denials | 1.26 (0.91) | 1.58 (0.97) | 1.59 (0.92) | 1.57 (0.94) |
| Average percent of handgun sales that are pawn redemptions | 0.52 (2.30) | 0.79 (3.33) | 0.86 (3.86) | 0.80 (3.22) |
| Average percent of handgun sales that are pawns | 0.39 (2.74) | 0.75 (4.01) | 0.77 (3.99) | 0.78 (4.14) |
| Average percent of sales in calendar year that become crime guns that year | 0.10 (0.17) | 0.15 (0.22) | 0.15 (0.20) | 0.15 (0.22) |
| Percent sales to police in the past year |  |  |  |  |
| Missing | 156,450 (3.31) | 1358 (3.53) | 138 (3.51) | 836 (3.42) |
| ≤5% | 1,248,111 (26.38) | 8461 (22.01) | 940 (23.89) | 5222 (21.38) |
| >5-10% | 1,867,441 (39.48) | 17,794 (46.29) | 1780 (45.24) | 11,362 (46.52) |
| >10-20% | 907,222 (19.18) | 7827 (20.36) | 776 (19.72) | 5150 (21.09) |
| >20% | 551,454 (11.66) | 3001 (7.81) | 301 (7.65) | 1854 (7.59) |
| **Characteristics of the firearm** |  |  |  |  |
| Low-cost manufacturers | 100,166 (2.12) | 2438 (6.34) | 257 (6.53) | 1625 (6.65) |
| Semiautomatic | 3,805,352 (81.45) | 33,370 (86.82) | 3465 (88.06) | 21,318 (87.30) |
| Caliber size |  |  |  |  |
| Small | 499,685 (10.57) | 2978 (7.75) | 276 (7.01) | 1976 (8.09) |
| Medium | 2,426,468 (51.34) | 19,237 (50.05) | 2067 (52.53) | 11,973 (49.03) |
| Large | 1,800,019 (38.09) | 16,223 (42.21) | 1592 (40.46) | 10,472 (42.88) |
| Any previous law enforcement holds | 64,862 (1.37) | 1230 (3.20) | 129 (3.28) | 695 (2.85) |
| Any previous consignment | 113,400 (2.40) | 441 (1.15) | 60 (1.52) | 284 (1.16) |
| Any previous intrafamilial transfer | 14,789 (0.31) | 55 (0.14) | 5 (0.13) | 43 (0.18) |
| Any previous law enforcement release | 26,840 (0.57) | 694 (1.81) | 67 (1.70) | 455 (1.86) |
| Any previous lost | 4958 (0.10) | 349 (0.91) | 32 (0.81) | 231 (0.95) |
| Any previous pawn redemption | 35,700 (0.75) | 486 (1.26) | 56 (1.42) | 309 (1.27) |
| Any previous pawn | 31,586 (0.67) | 441 (1.15) | 48 (1.22) | 281 (1.15) |
| Any previous stolen | 31,853 (0.67) | 6858 (17.84) | 579 (14.71) | 4266 (17.47) |
| **Characteristics of the purchaser-firearm pair^2^** |  |  |  |  |
| Not in possession of handgun | 51,047 (0.98) | 7238 (18.83) | 613 (15.58) | 4520 (18.51) |
| **Characteristic of the purchaser, while in possession of handgun^2^** |  |  |  |  |
| Age | 43.45 (13.75) | 35.54 (12.63) | 35.49 (12.15) | 35.11 (12.51) |
| Female sex | 552,234 (10.71) | 4526 (14.51) | 502 (15.11) | 2947 (14.81) |
| Citizen status | 5,056,858 (98.05) | 30,255 (96.96) | 3208 (96.57) | 19337 (97.16) |
| Foreign born | 763,734 (14.81) | 4154 (13.31) | 494 (14.87) | 2476 (12.44) |
| Race and ethnicity |  |  |  |  |
| Asian | 550,768 (10.68) | 2195 (7.03) | 257 (7.74) | 1319 (6.63) |
| Black | 218,732 (4.24) | 5154 (16.52) | 600 (18.06) | 3552 (17.85) |
| Hispanic | 888,950 (17.24) | 9101 (29.17) | 989 (29.77) | 5838 (29.33) |
| Native American / Pacific Islander | 79,821 (1.55) | 642 (2.06) | 61 (1.84) | 446 (2.24) |
| Other | 54,910 (1.06) | 444 (1.42) | 55 (1.66) | 277 (1.39) |
| White | 3,364,364 (65.23) | 13,666 (43.80) | 1360 (40.94) | 8471 (42.56) |
| Handguns bought in the last year |  |  |  |  |
| 0 | 3,646,133 (70.69) | 25,856 (82.86) | 2836 (85.37) | 16,407 (82.43) |
| 1 | 685,033 (13.28) | 3128 (10.02) | 285 (8.58) | 2011 (10.10) |
| 2-5 | 574,720 (11.14) | 1596 (5.11) | 162 (4.88) | 997 (5.00) |
| 6-12 | 166,912 (3.24) | 327 (1.05) | 34 (1.02) | 220 (1.11) |
| >12 | 85,062 (1.65) | 296 (0.95) | 5 (0.15) | 269 (1.35) |
| First handgun purchase | 2,239,096 (43.41) | 19,711 (63.17) | 2221 (66.86) | 12,419 (62.39) |
| Criminal history, arrests |  |  |  |  |
| Infraction with no other charge, past 10 years | 7203 (0.14) | 303 (0.97) | 37 (1.11) | 219 (1.10) |
| Alcohol intoxication, past 10 years | 218,535 (4.24) | 3557 (11.40) | 372 (11.20) | 2283 (11.47) |
| Firearm-related, past 10 years | 32,513 (0.63) | 963 (3.09) | 95 (2.86) | 659 (3.31) |
| Major violent crime, past 10 years | 42,648 (0.83) | 1243 (3.98) | 148 (4.46) | 848 (4.26) |
| Major property crime, past 10 yrs. | 23,439 (0.45) | 896 (2.87) | 92 (2.77) | 631 (3.17) |
| Distance to dealer |  |  |  |  |
| Missing | 695,284 (13.48) | 2044 (6.55) | 259 (7.80) | 1271 (6.39) |
| ≤5 miles | 1,438,540 (27.89) | 10,550 (33.81) | 1103 (33.20) | 6769 (34.01) |
| 5-20 miles | 2,037,057 (39.49) | 13,706 (43.93) | 1421 (42.78) | 8730 (43.86) |
| >20 miles | 986,979 (19.14) | 4903 (15.71) | 539 (16.23) | 3134 (15.75) |
| Purchaser geographic characteristics |  |  |  |  |
| SVI^1^, socioeconomic status | 44.87 (26.03) | 57.60 (26.99) | 58.30 (26.76) | 58.81 (26.79) |
| SVI, housing status | 52.34 (27.99) | 57.52 (28.14) | 57.31 (28.45) | 58.91 (27.96) |
| SVI, racial and ethnic minority status | 41.02 (25.92) | 53.97 (27.39) | 54.68 (26.85) | 54.63 (27.36) |
| SVI, housing type and transportation | 43.39 (27.59) | 52.66 (27.89) | 53.9 (27.98) | 52.92 (27.66) |
| RUCA^1^ code 1 | 4,295,119 (83.28) | 28,046 (89.88) | 3001 (90.34) | 17,846 (89.66) |
| **Characteristic of the purchaser, while *not* in possession of handgun^2^** |  |  |  |  |
| Age | 38.42 (13.49) | 36.45 (12.46) | 36.66 (12.42) | 36.26 (12.41) |
| Female sex | 6757 (13.24) | 1029 (14.22) | 84 (13.70) | 650 (14.38) |
| Citizen status | 49,820 (97.62) | 7049 (97.39) | 593 (96.74) | 4403 (97.41) |
| Foreign born | 6840 (13.40) | 992 (13.71) | 108 (17.62) | 607 (13.43) |
| Race and ethnicity |  |  |  |  |
| Asian | 3640 (7.13) | 597 (8.25) | 63 (10.28) | 389 (8.61) |
| Black | 5619 (11.01) | 1122 (15.50) | 98 (15.99) | 749 (16.57) |
| Hispanic | 11,257 (22.06) | 1759 (24.30) | 163 (26.59) | 1045 (23.12) |
| Native American / Pacific Islander | 923 (1.81) | 137 (1.89) | 19 (3.10) | 86 (1.90) |
| Other | 579 (1.13) | 100 (1.38) | 8 (1.31) | 68 (1.50) |
| White | 29,016 (56.86) | 3523 (48.67) | 262 (42.74) | 2183 (48.30) |
| Handguns bought in the last year |  |  |  |  |
| 0 | 38701 (75.81) | 5748 (79.41) | 499 (81.40) | 3594 (79.51) |
| 1 | 6883 (13.48) | 945 (13.06) | 70 (11.41) | 599 (13.25) |
| 2-5 | 4354 (8.53) | 482 (6.66) | 42 (6.85) | 285 (6.31) |
| 6-12 | 728 (1.43) | 44 (0.61) | 2 (0.33) | 30 (0.66) |
| >12 | 381 (0.75) | 19 (0.26) | 0 (0.00) | 12 (0.27) |
| First handgun purchase | 24,289 (47.58) | 3873 (53.51) | 350 (57.10) | 2408 (53.27) |
| Criminal history, arrests |  |  |  |  |
| Infraction with no other charge, past 10 years | 204 (0.40) | 36 (0.50) | 4 (0.65) | 21 (0.46) |
| Alcohol intoxication, past 10 years | 4608 (9.03) | 642 (8.87) | 50 (8.16) | 397 (8.78) |
| Firearm-related, past 10 years | 1558 (3.05) | 151 (2.09) | 6 (0.98) | 94 (2.08) |
| Major violent crime, past 10 years | 2079 (4.07) | 200 (2.76) | 15 (2.45) | 126 (2.79) |
| Major property crime, past 10 yrs. | 990 (1.94) | 130 (1.80) | 13 (2.12) | 78 (1.73) |
| Distance to dealer |  |  |  |  |
| Missing | 13,639 (26.72) | 1430 (19.76) | 132 (21.53) | 864 (19.12) |
| ≤5 miles | 13,113 (25.69) | 2104 (29.07) | 196 (31.97) | 1318 (29.16) |
| 5-20 miles | 16,526 (32.37) | 2640 (36.47) | 204 (33.28) | 1645 (36.39) |
| >20 miles | 7769 (15.22) | 1064 (14.70) | 81 (13.21) | 693 (15.33) |
| *Purchaser geographic characteristics* |  |  |  |  |
| SVI, socioeconomic status | 53.82 (27.52) | 55.52 (26.99) | 57.76 (27.68) | 55.52 (26.79) |
| SVI, housing status, per 10 units | 58.90 (28.37) | 57.88 (28.57) | 58.21 (29.36) | 58.81 (28.55) |
| SVI, racial and ethnic minority status | 47.13 (27.25) | 51.75 (26.60) | 55.07 (25.71) | 51.49 (26.77) |
| SVI, housing type and transportation | 49.45 (27.84) | 50.14 (27.83) | 53.45 (27.37) | 50.2 (27.63) |
| RUCA code 1 | 43,209 (84.65) | 6498 (89.78) | 552 (90.05) | 4047 (89.54) |

^1^ Abbreviations: SD: Standard Deviation, SVI: Social Vulnerability Index, RUCA: Rural Urban Commuting Area.

^2^ Numbers are mean (SD) or n (%) of firearms transactions.

## **Supplemental Table 3**. The associations of race and ethnicity with crime guns among low (<75^th^ percentile) vs high (≥75^th^ percentile) neighborhood racial and ethnic minority status (social vulnerability index subscale) and purchaser possession, among handguns^1^

|  | **All crime guns** | **Handguns picked up in violent crimes** | **Handguns picked up in weapons crimes** |
| --- | --- | --- | --- |
| **Description** | **Hazard Ratio (95% CI)** | **Hazard Ratio (95% CI)** | **Hazard Ratio (95% CI)** |
| **Possesses gun, neighborhood racial and ethnic minority status is high** | | | |
| Asian vs white | 0.74 (0.68, 0.81) | 0.90 (0.69, 1.17) | 0.70 (0.62, 0.78) |
| Black vs white | 3.52 (3.28, 3.77) | 3.52 (2.84, 4.38) | 3.83 (3.52, 4.17) |
| Hispanic vs white | 1.31 (1.24, 1.40) | 1.35 (1.12, 1.63) | 1.29 (1.20, 1.39) |
| Native American or Pacific Islander vs white | 1.44 (1.22, 1.70) | 1.08 (0.60, 1.94) | 1.58 (1.30, 1.93) |
| Other vs white | 1.35 (1.11, 1.64) | 2.26 (1.42, 3.61) | 1.13 (0.87, 1.47) |
| **Possesses gun, neighborhood racial and ethnic minority status is low** | | | |
| Asian vs white | 0.89 (0.84, 0.94) | 0.97 (0.82, 1.15) | 0.89 (0.83, 0.96) |
| Black vs white | 3.84 (3.69, 3.99) | 4.60 (4.09, 5.16) | 4.17 (3.97, 4.38) |
| Hispanic vs white | 1.48 (1.43, 1.54) | 1.58 (1.42, 1.76) | 1.50 (1.44, 1.57) |
| Native American or Pacific Islander vs white | 1.55 (1.41, 1.69) | 1.57 (1.18, 2.09) | 1.71 (1.53, 1.91) |
| Other vs white | 1.59 (1.42, 1.77) | 1.67 (1.19, 2.33) | 1.68 (1.47, 1.93) |
| **Does not possess gun, neighborhood racial and ethnic minority status is high** | | | |
| Asian vs white | 1.19 (0.97, 1.45) | 2.23 (1.09, 4.56) | 1.23 (0.96, 1.60) |
| Black vs white | 1.17 (1.00, 1.38) | 2.05 (1.10, 3.80) | 1.22 (0.99, 1.49) |
| Hispanic vs white | 1.00 (0.87, 1.16) | 1.84 (1.05, 3.24) | 0.98 (0.82, 1.18) |
| Native American or Pacific Islander vs white | 1.51 (1.05, 2.18) | 3.76 (1.26, 11.21) | 1.41 (0.86, 2.29) |
| Other vs white | 0.93 (0.60, 1.46) | 1.70 (0.38, 7.56) | 0.98 (0.56, 1.70) |
| **Does not possess gun, neighborhood racial and ethnic minority status is low** | | | |
| Asian vs white | 1.26 (1.12, 1.40) | 1.85 (1.31, 2.60) | 1.34 (1.16, 1.54) |
| Black vs white | 1.25 (1.14, 1.30) | 1.38 (1.01, 1.88) | 1.35 (1.21, 1.51) |
| Hispanic vs white | 1.11 (1.03, 1.21) | 1.29 (0.98, 1.70) | 1.06 (0.95, 1.17) |
| Native American or Pacific Islander vs white | 0.95 (0.76, 1.19) | 2.15 (1.25, 3.72) | 0.92 (0.69, 1.22) |
| Other vs white | 1.13 (0.88, 1.46) | 0.73 (0.23, 2.27) | 1.27 (0.94, 1.72) |

^1^ Model contains the same variables as found in Table 1 with the addition of a three-way interaction term between neighborhood racial and ethnic minority status, race and ethnicity, and firearm possession. Variables were entered into a Cox proportional hazard model which accounted for left truncation and clustering between transactions of the same firearm-purchaser pair, the firearm, the purchaser, the dealership the firearm was purchased at, and the purchaser’s and dealership’s census tracts.

## **Supplemental Table 4**. The associations of race and ethnicity with crime guns among purchasers with and without arrests for infractions with no other charge in the past 10 years and purchaser possession, among handguns^1^

|  | **All crime guns** | **Handguns picked up in violent crimes** | **Handguns picked up in weapons crimes** |
| --- | --- | --- | --- |
| **Description** | **Hazard Ratio (95% CI)** | **Hazard Ratio (95% CI)** | **Hazard Ratio (95% CI)** |
| **Possesses gun, has an infraction with no other charge in the past 10 years** | | | |
| Asian vs white | 1.76 (0.93, 3.34) | 3.93 (1.12, 13.82) | 1.29 (0.51, 3.23) |
| Black vs white | 5.69 (4.09, 7.92) | 5.18 (2.05, 13.13) | 7.22 (4.80, 10.85) |
| Hispanic vs white | 1.60 (1.14, 2.23) | 1.47 (0.58, 3.73) | 1.98 (1.31, 3.00) |
| Native American or Pacific Islander vs white | 1.90 (0.68, 5.32) | 0.00 (0.00, 0.00) | 3.16 (1.10, 9.06) |
| Other vs white | 2.61 (1.10, 6.20) | 4.18 (0.52, 33.85) | 3.33 (1.26, 8.78) |
| **Possesses gun, no infraction with no other charge in the past 10 years** | | | |
| Asian vs white | 0.83 (0.79, 0.87) | 0.93 (0.81, 1.08) | 0.81 (0.76, 0.87) |
| Black vs white | 3.70 (3.58, 3.84) | 4.23 (3.81, 4.70) | 4.03 (3.86, 4.21) |
| Hispanic vs white | 1.43 (1.38, 1.47) | 1.51 (1.37, 1.66) | 1.43 (1.38, 1.49) |
| Native American or Pacific Islander vs white | 1.51 (1.40, 1.64) | 1.45 (1.12, 1.88) | 1.67 (1.52, 1.84) |
| Other vs white | 1.51 (1.37, 1.66) | 1.83 (1.39, 2.40) | 1.50 (1.33, 1.70) |
| **Does not possess gun, has an infraction with no other charge in the past 10 years** | | | |
| Asian vs white | 0.00 (0.00, 0.01) | 0.00 (0.00, 0.02) | 0.00 (0.00, 0.02) |
| Black vs white | 1.38 (0.53, 3.60) | 1.69 (0.15, 19.23) | 2.84 (0.58, 13.94) |
| Hispanic vs white | 1.16 (0.44, 3.04) | 0.75 (0.05, 12.03) | 2.96 (0.62, 14.01) |
| Native American or Pacific Islander vs white | 1.08 (0.12, 9.45) | 0.00 (0.00, 0.00) | 3.72 (0.31, 44.23) |
| Other vs white | 0.00 (0.00, 0.00) | 0.00 (0.00, 0.00) | 0.00 (0.00, 0.02) |
| **Does not possess gun, no infraction with no other charge in the past 10 years** | | | |
| Asian vs white | 1.19 (1.08, 1.32) | 1.66 (1.23, 2.26) | 1.27 (1.11, 1.44) |
| Black vs white | 1.19 (1.10, 1.29) | 1.37 (1.04, 1.79) | 1.27 (1.15, 1.41) |
| Hispanic vs white | 1.04 (0.97, 1.12) | 1.26 (1.00, 1.60) | 1.00 (0.92, 1.10) |
| Native American or Pacific Islander vs white | 1.05 (0.87, 1.28) | 2.28 (1.41, 3.70) | 0.99 (0.77, 1.27) |
| Other vs white | 1.07 (0.86, 1.33) | 0.86 (0.36, 2.10) | 1.17 (0.90, 1.54) |

^1^ Model contains the same variables as found in Table 1 with the addition of a three-way interaction term between past infraction-only arrest, race and ethnicity, and firearm possession. Variables were entered into a Cox proportional hazard model which accounted for left truncation and clustering between transactions of the same firearm-purchaser pair, the firearm, the purchaser, the dealership the firearm was purchased at, and the purchaser’s and dealership’s census tracts.

## **Supplemental Table 5**. Characteristics supplied by the dealer, firearm, and purchaser across a long gun being picked up in a crime

|  | **Never a crime gun, n=2,658,863** | **All crime guns, n=6806** |
| --- | --- | --- |
| **Characteristic** | Mean (SD^1^) or n (%) | Mean (SD) or n (%,) |
|  |  |  |
| **Characteristics supplied by dealer** |  |  |
| Average sales per year, per 1000 | 2.17 (1.92) | 2.18 (1.92) |
| Average percent denials | 1.10 (1.14) | 1.30 (0.84) |
| Average percent of handgun sales that are pawn redemptions | 0.45 (1.96) | 0.54 (2.39) |
| Average percent of handgun sales that are pawns | 0.37 (2.50) | 0.47 (3.04) |
| Average percent of sales in calendar year that become crime guns that year | 0.09 (0.17) | 0.12 (0.18) |
| Percent sales to police in the past year | 8.59 (10.77) | 7.14 (7.29) |
| Missing | 156,942 (5.64) | 571 (8.39) |
| ≤5% | 1,005,130 (36.12) | 2593 (38.10) |
| >5-10% | 1,037,601 (37.29) | 2606 (38.29) |
| >10-20% | 410,750 (14.76) | 771 (11.33) |
| >20% | 172,025 (6.18) | 265 (3.89) |
| **Characteristics of the firearm** |  |  |
| Semiautomatic | 1,532,605 (55.08) | 3503 (51.47) |
| Caliber size |  |  |
| Rifle, rimfire | 370,615 (13.32) | 1129 (16.59) |
| Rifle, frame/receiver only | 469,867 (16.89) | 205 (3.01) |
| Rifle, centerfire | 1,168,450 (41.99) | 3031 (44.53) |
| Shotgun, not 410 | 773,516 (27.80) | 2441 (35.87) |
| Any previous law enforcement holds | 13,811 (0.50) | 97 (1.43) |
| Any previous consignment | 21,442 (0.77) | 49 (0.72) |
| Any previous intrafamilial transfer | 3062 (0.11) | 19 (0.28) |
| Any previous law enforcement release | 4428 (0.16) | 41 (0.60) |
| Any previous lost | 368 (0.01) | 9 (0.13) |
| Any previous pawn redemption | 11,532 (0.41) | 46 (0.68) |
| Any previous pawn | 9665 (0.35) | 37 (0.54) |
| Any previous stolen | 4652 (0.17) | 319 (4.69) |
| **Characteristics of the purchaser-firearm pair^2^** |  |  |
| Not in possession of handgun | 5957 (0.20) | 331 (4.81) |
| **Characteristic of the purchaser, while in possession of handgun^2^** |  |  |
| Age | 42.93 (14.07) | 36.16 (13.28) |
| Female sex | 169,486 (5.93) | 669 (10.33) |
| Citizen status | 2,793,574 (97.81) | 6262 (96.67) |
| Foreign born | 404,543 (14.16) | 928 (14.33) |
| Race and ethnicity |  |  |
| Asian | 290,885 (10.18) | 554 (8.55) |
| Black | 79,851 (2.80) | 343 (5.29) |
| Hispanic | 505,216 (17.69) | 1993 (30.77) |
| Native American/Pacific Islander | 48,233 (1.69) | 137 (2.11) |
| Other | 31,512 (1.10) | 107 (1.65) |
| White | 190,0387 (66.54) | 3344 (51.62) |
| Handguns bought in the last year |  |  |
| 0 | 2,130,321 (74.6) | 5360 (82.7) |
| 1 | 322,502 (11.3) | 464 (7.2) |
| 2-5 | 296,100 (10.4) | 444 (6.9) |
| 6-12 | 77,607 (2.7) | 160 (2.5) |
| >12 | 29,554 (1.0) | 50 (0.8) |
| Only purchased long guns | 908,707 (31.8) | 2928 (45.2) |
| Criminal history, arrests |  |  |
| Infraction with no other charge, past 10 years | 3665 (0.13) | 34 (0.52) |
| Alcohol intoxication, past 10 years | 129,823 (4.55) | 721 (11.13) |
| Firearm-related, past 10 years | 16,306 (0.57) | 145 (2.24) |
| Major violent crime, past 10 years | 20,381 (0.71) | 226 (3.49) |
| Major property crime, past 10 years | 10,629 (0.37) | 146 (2.25) |
| Distance to dealer |  |  |
| Missing | 365,591 (12.80) | 396 (6.11) |
| ≤5 miles | 850,249 (29.77) | 2364 (36.49) |
| 5-20 miles | 1,118,151 (39.15) | 2816 (43.47) |
| >20 miles | 522,093 (18.28) | 902 (13.92) |
| Purchaser geographic characteristics |  |  |
| SVI^1^, socioeconomic status | 44.99 (26.00) | 56.59 (26.92) |
| SVI, housing | 52.92 (27.96) | 55.40 (28.04) |
| SVI, racial and ethnic minority | 40.62 (26.08) | 52.53 (28.05) |
| SVI, housing type, transportation | 43.41 (27.48) | 50.61 (27.90) |
| RUCA^1^ code 1 | 2,315,932 (81.09) | 5677 (87.64) |
| **Characteristic of the purchaser, while *not* in possession of handgun^2^** |  |  |
| Age | 38.31 (14.56) | 35.79 (13.67) |
| Female sex | 556 (9.55) | 25 (7.62) |
| Citizen status | 5662 (97.24) | 319 (97.26) |
| Foreign born | 742 (12.74) | 56 (17.07) |
| Race and ethnicity |  |  |
| Asian | 479 (8.23) | 32 (9.76) |
| Black | 301 (5.17) | 16 (4.88) |
| Hispanic | 1308 (22.46) | 90 (27.44) |
| Native American/Pacific Islander | 163 (2.80) | 7 (2.13) |
| Other | 82 (1.41) | 10 (3.05) |
| White | 3490 (59.93) | 173 (52.74) |
| Handguns bought in the last year |  |  |
| 0 | 4500 (77.3) | 264 (80.5) |
| 1 | 666 (11.4) | 36 (11.0) |
| 2-5 | 553 (9.5) | 23 (7.0) |
| 6-12 | 66 (1.1) | 4 (1.2) |
| >12 | 38 (0.7) | 1 (0.3) |
| Only purchased long guns | 2022 (34.7) | 133 (40.5) |
| Criminal history, arrests |  |  |
| Infraction with no other charge, past 10 years | 12 (0.21) | 0 (0.00) |
| Alcohol intoxication, past 10 years | 492 (8.45) | 29 (8.84) |
| Firearm-related, past 10 years | 146 (2.51) | 11 (3.35) |
| Major violent crime, past 10 years | 196 (3.37) | 6 (1.83) |
| Major property crime, past 10 years | 100 (1.72) | 5 (1.52) |
| Distance to dealer |  |  |
| Missing | 358 (6.15) | 17 (5.18) |
| ≤5 miles | 2049 (35.19) | 127 (38.72) |
| 5-20 miles | 2324 (39.91) | 144 (43.90) |
| >20 miles | 1092 (18.75) | 40 (12.20) |
| *Purchaser geographic characteristics* |  |  |
| SVI, socioeconomic status | 53.51 (27.32) | 55.00 (27.31) |
| SVI, housing | 58.94 (28.57) | 57.95 (27.65) |
| SVI, racial and ethnic minority status | 44.87 (27.10) | 48.69 (27.06) |
| SVI, housing type and transportation | 49.43 (27.78) | 48.51 (28.52) |
| RUCA code 1 | 4566 (78.41) | 272 (82.93) |

^1^ Abbreviations: SD: Standard Deviation, SVI: Social Vulnerability Index, RUCA: Rural Urban Commuting Area.

^2^ Numbers are mean (SD) or n (%) of firearms transactions.

## **Supplemental Table 6**. Multivariable adjusted characteristics associated with a long gun being picked up in a crime^1^

|  | **All crime guns** | | |
| --- | --- | --- | --- |
| **Characteristic** | **Hazard Ratio (95% CI^2^)** | **Chi-Square** | **P-value** |
|  |  |  |  |
| **Characteristics supplied by dealer** |  |  |  |
| Average percent denials | 1.01 (1.01, 1.02) | 54.9 | <0.0001 |
| Average percent of handgun sales that are pawn redemptions | 1.03 (1.02, 1.04) | 16.3 | <0.0001 |
| Average percent of handgun sales that are pawns | 0.98 (0.97, 1.00) | 7.5 | 0.006 |
| Average percent of sales in calendar year that become crime guns that year | 1.31 (1.25, 1.37) | 131.7 | <0.0001 |
| Percent sales to police in the past year |  |  |  |
| Missing (dealership not open) | 1.14 (1.02, 1.27) | 4.9 | 0.03 |
| ≤5% | 1.12 (1.03, 1.22) | 7.5 | 0.006 |
| >5-10% | 1.09 (1.00, 1.18) | 3.8 | 0.05 |
| >10-20% | Reference | | |
| >20% | 1.00 (0.87, 1.15) | 0.0 | 0.98 |
|  |  |  |  |
| **Characteristics of the firearm** |  |  |  |
| Semiautomatic | 1.11 (1.05, 1.17) | 16.1 | <0.0001 |
| Caliber size |  |  |  |
| Rifle, centerfire | Reference | | |
| Rifle, frame/receiver only | 0.16 (0.14, 0.18) | 628.7 | <0.0001 |
| Rifle, rimfire | 1.13 (1.05, 1.21) | 11.2 | 0.0008 |
| Shotgun, not 410 | 1.20 (1.13, 1.27) | 41.8 | <0.0001 |
| Any previous consignment | 1.47 (1.11, 1.96) | 7.0 | 0.008 |
| Any previous intrafamilial transfer | 2.07 (1.27, 3.35) | 8.6 | 0.003 |
| Any previous lost | 6.31 (2.83, 14.07) | 20.3 | <0.0001 |
| Any previous stolen | 17.43 (8.69, 34.94) | 64.8 | <0.0001 |
|  |  |  |  |
| **Characteristics of the purchaser** |  |  |  |
| ***In possession of long gun*** |  |  |  |
| Age, per 10 years | 0.75 (0.73, 0.76) | 682.9 | <0.0001 |
| Female sex | 1.70 (1.57, 1.85) | 159.3 | <0.0001 |
| Citizen status | 0.77 (0.66, 0.9) | 11.2 | 0.0008 |
| Foreign born | 0.98 (0.9, 1.06) | 0.3 | 0.61 |
| Race and ethnicity |  |  |  |
| Asian | 0.92 (0.83, 1.01) | 2.9 | 0.09 |
| Black | 1.82 (1.63, 2.04) | 106.6 | <0.0001 |
| Hispanic | 1.25 (1.17, 1.33) | 41.6 | <0.0001 |
| Native American/Pacific Islander | 1.24 (1.04, 1.48) | 5.9 | 0.02 |
| Other | 1.52 (1.25, 1.85) | 17.9 | <0.0001 |
| White | Reference | | |
| Handguns bought in the last year |  |  |  |
| 0 | Reference | | |
| 1 | 0.80 (0.72, 0.88) | 19.6 | <0.0001 |
| 2-5 | 0.95 (0.86, 1.05) | 1.0 | 0.31 |
| 6-12 | 1.55 (1.32, 1.82) | 27.9 | <0.0001 |
| >12 | 1.50 (1.13, 2.00) | 7.8 | 0.005 |
| Only purchased long guns | 1.20 (1.13, 1.26) | 40.4 | <0.0001 |
| Criminal history, arrests |  |  |  |
| Infraction with no other charge, past 10 years | 1.75 (1.24, 2.46) | 10.1 | 0.002 |
| Alcohol intoxication, past 10 years | 1.96 (1.81, 2.12) | 269.1 | <0.0001 |
| Firearm-related, past 10 years | 1.93 (1.58, 2.35) | 42.5 | <0.0001 |
| Major violent crime, past 10 years | 2.62 (2.23, 3.08) | 137.8 | <0.0001 |
| Major property crime, past 10 yrs. | 2.57 (2.15, 3.08) | 105.3 | <0.0001 |
| Distance to dealer |  |  |  |
| Missing | 0.76 (0.68, 0.84) | 25.5 | <0.0001 |
| ≤5 miles | 1.04 (0.99, 1.10) | 2.1 | 0.15 |
| 5-20 miles | Reference | | |
| >20 miles | 0.76 (0.70, 0.82) | 51.8 | <0.0001 |
| Purchaser geographic characteristics |  |  |  |
| SVI^2^, socioeconomic status, per 10 units | 1.12 (1.10, 1.14) | 196.9 | <0.0001 |
| SVI, housing | 0.97 (0.96, 0.98) | 35.7 | <0.0001 |
| SVI, racial and ethnic minority status, per 10 units | 1.05 (1.03, 1.06) | 37.9 | <0.0001 |
| RUCA^2^ code 1 | 1.45 (1.34, 1.57) | 80.1 | <0.0001 |
|  |  |  |  |
| ***Not in possession of long gun*** |  |  |  |
| Age, per 10 years | 0.91 (0.84, 1.00) | 4.3 | 0.04 |
| Female sex | 0.76 (0.50, 1.17) | 1.5 | 0.22 |
| Citizen status | 1.69 (0.80, 3.54) | 1.9 | 0.17 |
| Foreign born | 1.52 (1.07, 2.18) | 5.3 | 0.02 |
| Race and ethnicity |  |  |  |
| Asian | 1.02 (0.65, 1.61) | 0.0 | 0.93 |
| Black | 0.90 (0.53, 1.53) | 0.2 | 0.69 |
| Hispanic | 1.08 (0.79, 1.49) | 0.2 | 0.62 |
| Native American/Pacific Islander | 0.93 (0.43, 2.01) | 0.0 | 0.86 |
| Other | 1.79 (0.91, 3.53) | 2.9 | 0.09 |
| White | Reference | | |
| Handguns bought in the last year |  |  |  |
| 0 |  |  |  |
| 1 | 1.07 (0.74, 1.57) | 0.1 | 0.71 |
| 2-5 | 0.86 (0.54, 1.39) | 0.4 | 0.55 |
| 6-12 | 1.75 (0.65, 4.78) | 1.2 | 0.27 |
| >12 | 1.01 (0.15, 6.94) | 0.0 | 0.99 |
| Only purchased long guns | 1.18 (0.92, 1.52) | 1.8 | 0.18 |
| Criminal history, arrests |  |  |  |
| Infraction with no other charge, past 10 years | 0.00 (0.00, 0.00) | 479.4 | <0.0001 |
| Alcohol intoxication, past 10 years | 1.07 (0.73, 1.57) | 0.1 | 0.74 |
| Firearm-related, past 10 years | 1.64 (0.88, 3.07) | 2.4 | 0.12 |
| Major violent crime, past 10 years | 0.62 (0.27, 1.42) | 1.3 | 0.26 |
| Major property crime, past 10 yrs. | 0.92 (0.37, 2.30) | 0.0 | 0.87 |
| Distance to dealer, km |  |  |  |
| Missing | 1.16 (0.70, 1.93) | 0.3 | 0.57 |
| ≤5 miles | 1.00 (0.78, 1.29) | 0.0 | 0.97 |
| 5-20 miles | Reference | | |
| >20 miles | 0.64 (0.45, 0.92) | 5.9 | 0.02 |
| Purchaser geographic characteristics |  |  |  |
| SVI, socioeconomic status, per 10 units | 0.98 (0.92, 1.04) | 0.5 | 0.47 |
| SVI, housing | 0.99 (0.95, 1.04) | 0.1 | 0.76 |
| SVI, racial and ethnic minority status, per 10 units | 1.04 (0.98, 1.11) | 1.5 | 0.22 |
| RUCA code 1 | 1.00 (0.73, 1.37) | 0.0 | 0.98 |

^1^ All variables were entered into a Cox proportional hazard model which accounted for left truncation and clustering between transactions of the same firearm-purchaser pair, the firearm, the purchaser, the dealership the firearm was purchased at, and the purchaser’s and dealership’s census tracts.

^2^ Abbreviations: CI: Confidence Interval, SVI: Social Vulnerability Index, RUCA: Rural Urban Commuting Area
